# Direct sialic acid 4-OAc substitution by nitrogen, sulfur and carbon nucleophiles with retention of stereochemistry†

**DOI:** 10.1039/d2ra01576e

**Published:** 2022-04-19

**Authors:** Tiago Bozzola, Ulf J. Nilsson, Ulf Ellervik

**Affiliations:** Centre for Analysis and Synthesis, Department of Chemistry, Lund University P.O. Box 124 SE-221 00 Lund Sweden ulf.ellervik@chem.lu.se

## Abstract

A direct one-step nucleophilic substitution of the 4-OAc of acetyl protected Neu5Ac is presented. Previously published methods for direct substitution of the 4-OAc are limited to cyclic secondary amines. Here we present conditions that allow for a much wider range of nitrogen nucleophiles as well as thiols and cyanide, to be used. The present investigation significantly broadens the scope of 4-aminations and allows for the introduction of a wide variety of different nucleophiles.

## Abstract 2

A direct one-step nucleophilic substitution of the 4-OAc of acetyl protected Neu5Ac is presented.

## Graphical abstract

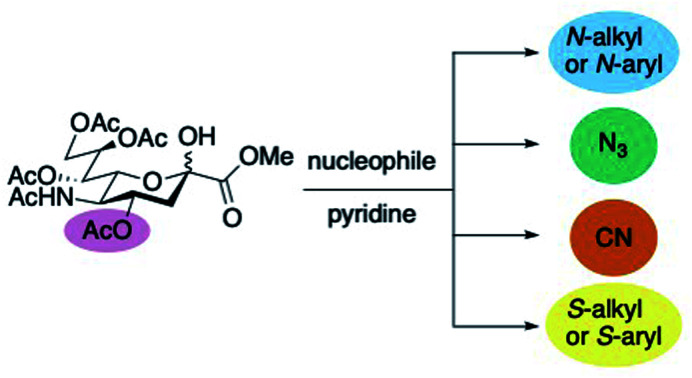

5-Acetylneuraminic acid (Neu5Ac) is the most abundant sialic acid, *i.e.* a member of a family of acidic 9-carbon sugars.^1^ Sialic acids are often found in the terminal positions of cell surface glycans and thus play crucial roles in both physiological and pathological conditions.^2,3^ Sialic acids are consequently important drug candidates and it is crucial to develop tools to functionalize and study unnatural sialic acid derivatives.^4^ As of now, modification of position 4 of sialic acid is complicated and examples in the literature are scarce.^5–7^ The introduction of an azido functionality has been achieved in the synthesis of the influenza drug Zanamivir, but such compounds still require long synthetic routes in the case of sialic acid derivatives other than glycals (Scheme 1).^8–12^

**Scheme 1:**
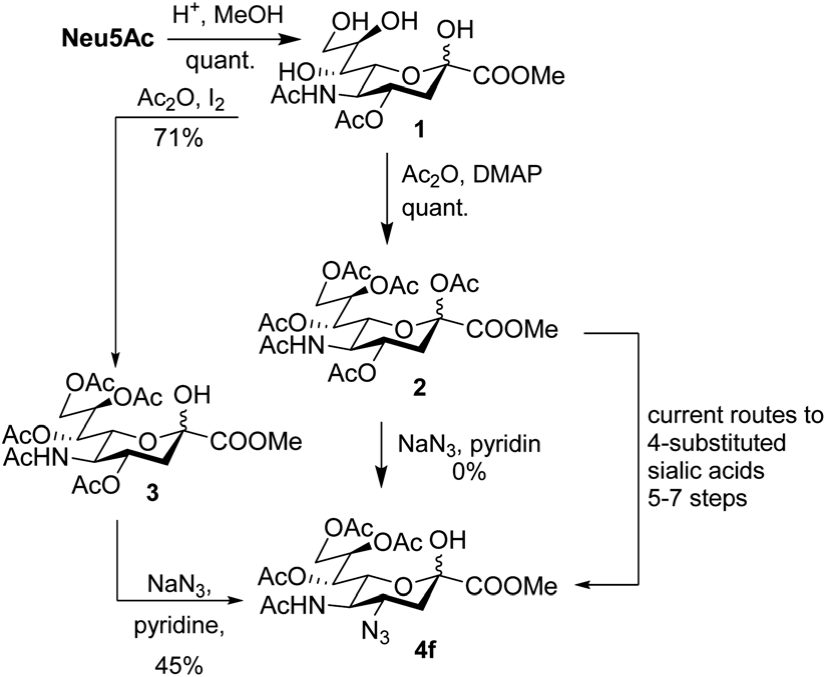
Synthesis of 4-amino sialic acids.

An elegant and convenient one-step amination was presented by Ye *et al.* with direct substitution of the 4-OAc of peracetylated Neu5Ac (2), by cyclic secondary amines with retention of stereochemistry.^13^ Compound 2 was stirred with 10 equivalents of the cyclic amine in pyridine for up to 48 hours and the stereochemical retention was hypothesized to result from neighbouring group participation by the 5-NHAc. However, the reported scope of the reaction remained limited to five- and six-membered secondary cyclic amines, such as morpholine and pyrrolidine. Acyclic, aromatic and hindered amines were reported as unreactive by the authors. Interestingly, Ye *et al.* showed that the reaction proceeded well using the hemiacetal 3. We hypothesized that the efficiency of the 4-amination reactions are proportional to the nucleophilicity of the amine, as cyclic amines are better nucleophiles compared to primary and acyclic secondary ones.^14^ Benzylamine, *N*-methylbenzylamine, aniline, and *N*-methylaniline were thus selected in an initial attempt to test this hypothesis. However, under the reported reaction conditions, the reaction with benzylamine led to a multitude of side products, including the partly deacetylated glycal 5 (Chart 1).

**Chart 1:**
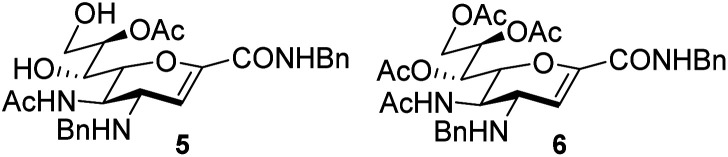
4-Aminations with benzylamine.

To prevent the observed side products, the reaction was instead conducted at −5 °C with 3 equivalents of benzylamine. Similar side products, such as 6 (Chart 1), were still formed, but we could also isolate 4a in 25% yield (Table 1). The reaction with *N*-methylbenzylamine yielded the desired 4b in 39% yield without further optimization. The reaction with aniline, as expected by the reduced nucleophilicity of the aromatic nitrogen, led to extended reaction time and formation of a mixture of side products. The reaction with *N*-methylaniline did not yield any product, neither through extended reaction time (>30 days), nor increased temperature (14 h at 80 °C, microwave reactor).

**Table 1:** 4-Amination reactions^a^

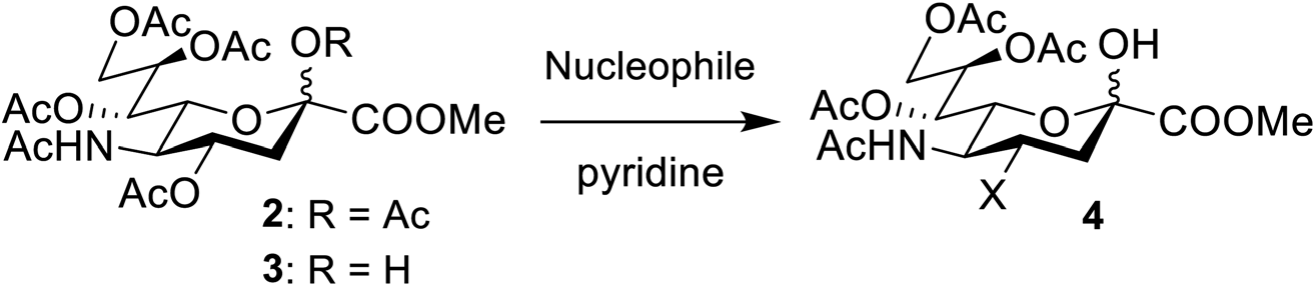					
Compound	X	R	Time	Temperature	Yield
4a	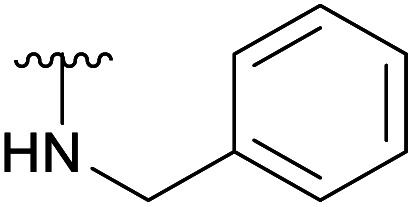	Ac	3 days	−5 °C	25%
		H	20 h	−10 °C	34%
4b	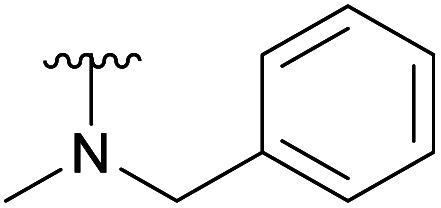	Ac	7 days	RT	39%
		H	—	—	Not tested
4c	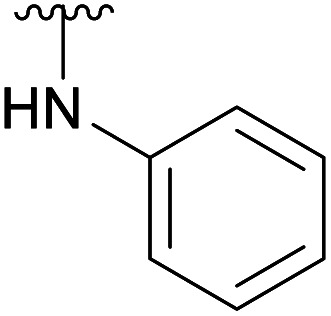	Ac	30 days	RT	34%
		H	17 days	RT	39%
4d	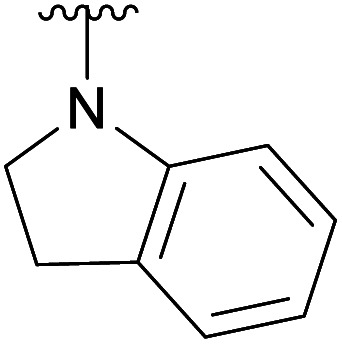	Ac	30 days	RT	66%
		H	—	—	Not tested
4e	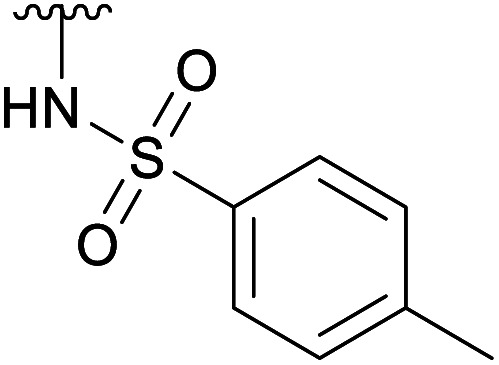	Ac	3 days^b^	RT	20%
		H	2 h^c^	RT	25%
4f	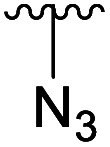	Ac	>30 days	RT	0%
		H	7 days	RT	45%
4g	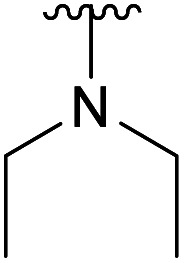	Ac	>30 days	RT	0%
		H	4 h	RT	41%
4h	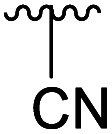	Ac	7 days	RT	21%
		H	2 days	RT	38%
4i	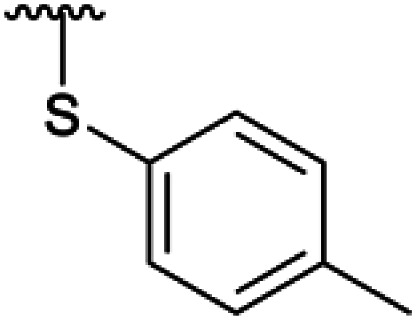	Ac	—	—	Not tested
		H	4 hours	RT	89%
4l	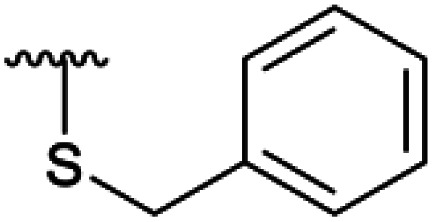	Ac	—	—	Not tested
		H	4 hours	RT	63%

a Reaction conditions: 3–10 eq of nucleophile in pyridine at room temperature until disappearance of starting material or appearance of side products.

b 3 eq of t-BuOK are additionally present.

c 3 eq of Cs_2_CO_3_ are additionally present.

To further investigate the scope of the reaction, other nitrogen containing nucleophiles such as acyclic amines, sulfonamide, as well as azide and cyanide ions were included (Table 1).

While the reaction with *p*-toluenesulfonamide and potassium cyanide proceeded to give 20% yield after 3 and 7 days respectively (Table 1, entries e and g), azide, and some acyclic amines did not result in product formation. Ye *et al.* proposed a reaction mechanism in which the 5-acetamide first reacts in an intramolecular nucleophilic substitution, with the 4-acetate as leaving group, to give the oxazolinium intermediate. This intermediate was supposed to be stabilized by the oxygen atom of the 2-OAc. The cyclic secondary amines then supposedly reacted with the activated 2-OAc to give the 2-OH. Finally, a second molecule of the amine reacted in an S_N_2-fashion with the oxazolinium moiety to give the product.

We instead argue that the reaction starts with a nucleophilic addition–elimination reaction that results in deacetylation of the 2-OAc and that this is the rate-limiting step (Scheme 2a). While some nitrogen nucleophiles, such as cyclic secondary amines, are capable both of nucleophilic addition–elimination and nucleophilic substitution, others, such as diethyl amine and azide ions are limited to the latter reaction. To test this hypothesis, we synthesized compound 3^15^ and re-examined the reaction. To our delight, the reaction with diethyl amine proceeded smoothly with full consumption of starting material, and 41% yield, in 4 hours (Table 1, entry g).

**Scheme 2:**
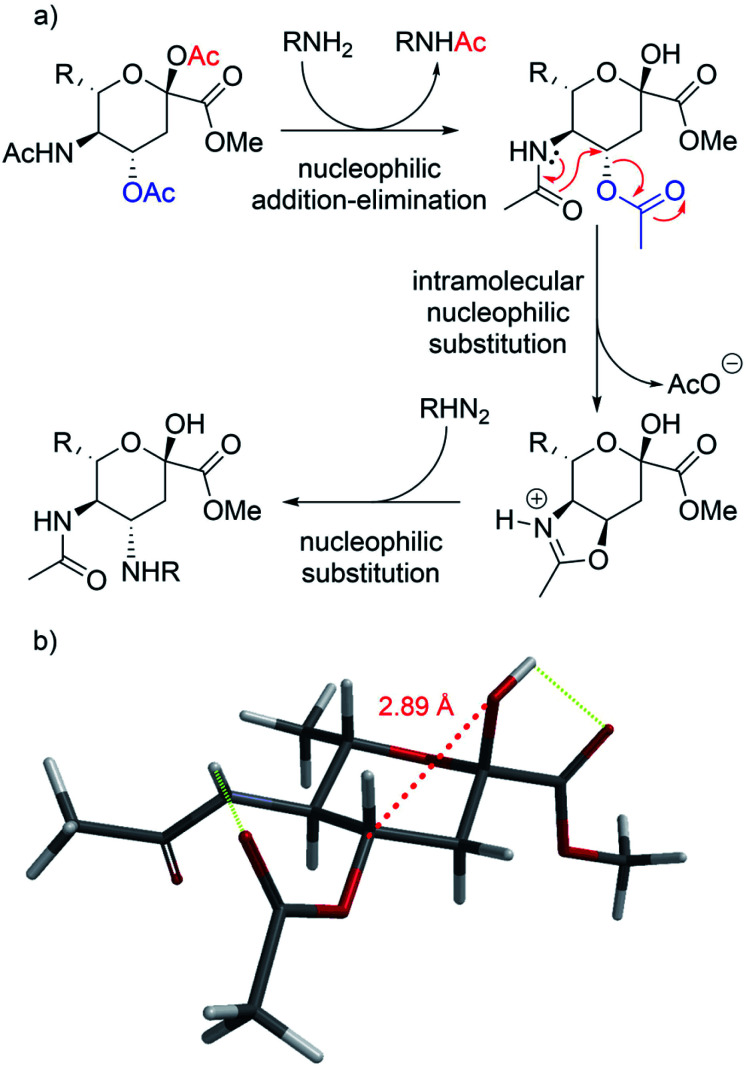
(a) Proposed mechanism of 4-aminations. (b) Stick model of compound 3 (the glycerol chain is omitted for clarity). In red the 02 to C4 distance, while in green the hydrogen bond between the proton on the 2-OH and the C1 carbonyl.

Subsequently, we tested the reactions previously attempted with 2. Although the yields remained moderate, the reactions proceeded fast and gave products even with azide, where no reaction was observed with 2. The possibility to insert an azide group at C4 of sialic acid opens up for a multitude of reactions, such as CuAAC Huisgen cycloadditions, whereas the cyanide addition selectively creates a carbon–carbon bond at C4 and facilitates insertion of methylene bridged amines and the formation of various heterocycles such as tetrazoles. At this point, we also included thiocresol and benzyl mercaptan to establish if the array of reacting nucleophiles also could include thiols. The reactions with thiols proceeded fast and in good yields to the desired products. This opens up for a multitude of new chemical entities, since the sulfides can be selectively oxidized to sulfoxides and sulfones.

We argue that the 2-OH, in contrast to the electron poor 2-OAc, enables the reaction by through–space interactions as previously reported by Miljković *et al.* (Scheme 2b).^16^ The 2-OH is positioned at a 2.87 Å from C4, the same distance previously reported between the substituent at C4 and the oxacarbenium ion in the case of d-galactopyranosides.^16^ The electron density on the 2-OH of compound 3 is accentuated by an internal hydrogen bond between the proton of the 2-OH and the carbonyl at C1 (Scheme 2b). Through space, the 2-OH is then able to donate into the antibonding orbital (σ*) of the C4–O4 bond, thus weakening the σ bond. Subsequentially, the HOMO from the carbonyl oxygen of the acetamide is capable of forming the oxazolinium ring, to later allow for the final S_N_2. Interestingly, the corresponding 2-OMe compound was unreactive *versus* cyanide ions (data not shown). An alternative explanation would be a reaction *via* the open-chain form, which is only accessible with the hemiacetal.

## Conclusions

Sialic acids are complex monosaccharide structures typically found at the terminal position of cell surface glycans and are important drug candidates. In order to functionalize and study unnatural sialic acid derivatives, access to efficient methods for derivatization of complex and poly-functional sialic acids is crucial.

As of now, synthesis of 4-amino sialic acid derivatives requires long synthetic routes. In our research for routes to 4-aminated sialic acid analogues, we investigated a convenient one-step amination with direct substitution of the 4-OAc of peracetylated Neu5Ac. To expand the scope of the reaction, which was limited to secondary cyclic amines, we investigated several nitrogen nucleophiles under various conditions and observed that, by using a 2-OH derivative as starting material, the scope was expanded to involve not only acyclic and aromatic amines, but also nucleophiles such as thiols, azide and cyanide ions.

Sulfide, azido and cyano functionalities allow for further derivatisation and are intensively used in drug discovery programs. The present investigation thus significantly broadens the scope of 4-substitutions and represents the shortest route to 4-aminated sialic acids, and opens up for efficient syntheses of other sialic acid derivatives *via* installation of and subsequent reaction of 4-N_3_,4-CN, or 4-SR groups.

## Experimental

### General information

All moisture-and air-sensitive reactions were carried out under an atmosphere of dry nitrogen using oven-dried glassware. All solvents were dried using an MBRAUN SPS-800 Solvent purification system before use unless otherwise stated. Purchased reagents were used without further purification. Thin-layer chromatography was performed on precoated TLC glass plates with silica gel 60 F254 0.25 mm (Merck). Spots were visualized with UV light or by charring with 10% H_2_SO_4_ in ethanol. Biotage Isolute phase separators were used for drying of combined organic layers. Preparative chromatography was performed on a Biotage Isolera One flash purification system using Biotage SNAP Sfär HD silica cartridges. Optical rotationswere measured on a Bellingham and Stanley model ADP450 polarimeter and are reported as [α]_D_^*T*^ (*c* = g/100 mL), where D indicates the sodium D line (589 nm) and *T* indicates the temperature. NMR spectra were recorded at ambient temperatures on a Bruker Avance II at 400 MHz (^1^H) and 100 MHz (^13^C) or a Bruker Ascend at 500 MHz (^1^H) and 125 MHz (^13^C) and assigned using 2D methods (COSY, HMQC). Chemical shifts are reported in ppm, with reference to residual solvent peaks (*δ*_H_ CHCl_3_ = 7.26 ppm, CD_3_OH = 3.31 ppm) and solvent signals (*δ*_C_ CDCl_3_ = 77.0 ppm, CD_3_OD = 49.0 ppm). Coupling constant values are given in Hz. Mass spectra were recorded on Waters XEVO G2 (positive ESI). Infrared spectroscopy was recorded on a Bruker *α* II FT-IR spectrometer. IR was only used to confirm structural features, and only the peak of interest was reported.

### General procedure for 4-amination

Starting material 2 or 3 was dissolved in dry pyridine, the mixture was allowed to attain the designated temperature, and the desired amine (3–10 eq.) was added. The reaction was monitored *via* TLC and LCMS until either full conversion, or appearance of side products. The reaction mixture was then evaporated, and the residues dissolved in EtOAc, washed with distilled water and brine, dried and purified by flash chromatography.

Methyl 5-acetamido-4-azido-7,8,9-tri-*O*-acetyl-3,4,5-trideoxy-d-glycero-β-d-galacto-non-2-ulopyranosidonate (4f) starting material 3 (1 g, 2.03 mmol) was reacted with sodium azide (0.396 g, 6.09 mmol). The reaction was completed after 7 days and the reaction mixture evaporated and flash chromatography performed directly (Hep : EtOAc 60 : 40 → 20 : 80 over 6 CV, then isocratic). The purification afforded 4f as an amorphous white solid (438 mg, 45%). [α]_D_^20^ + 18 (*c* 0.5, CH_2_Cl_2_). ^1^H NMR (500 MHz, CDCl_3_) *δ* 5.52 (d, *J* = 9.4 Hz, 1H, AcN-H), 5.32 (dd, *J* = 6.5, 2.0 Hz, 1H, H-7), 5.24 (td, *J* = 6.6, 2.4 Hz, 1H, H-8), 4.41 (dd, *J* = 12.4, 2.3 Hz, 1H, H-9), 4.30 (dd, *J* = 10.5, 2.0 Hz, 1H, H-6), 4.05 (m, 2H, H-9, H-4), 3.87 (s, 3H), 3.80–3.66 (m, 1H, H-5), 2.21 (dd, *J* = 13.1, 4.9 Hz, 1H, H-3_eq_), 2.15–1.99 (m, 13H, H-3_a*x*_, OCOCH_3_*x* 3, HNCOCH_3_). ^13^C NMR (126 MHz, CDCl_3_) *δ* 171.3, 170.9, 170.6, 169.2, 94.5, 70.5, 70.0, 68.0, 62.7, 58.0, 53.8, 51.1, 36.2, 23.6, 21.2, 21.1, 21.0, 20.9. ESI-MS (*m*/*z*): Calcd. for C_18_H_26_N_4_O_11_ [M + Na]^+^, 497.1496; found, 497.1490. IR: 2106 cm^−1^ (N_3_).

## Author contributions

Tiago Bozzola – conceptualization, investigation, visualization, writing – original draft, writing – review & editing. Ulf Nilsson – conceptualization, funding acquisition, supervision, visualization, writing – original draft, writing – review & editing. Ulf Ellervik – conceptualization, funding acquisition, supervision, visualization, writing – original draft, writing – review & editing.

## Conflicts of interest

There are no conflicts to declare.

## Supplementary Material

RA-012-D2RA01576E-s001
