# Supplementary material for: Direct sialic acid 4-OAc substitution by nitrogen, sulfur and carbon nucleophiles with retention of stereochemistry
Source: RSC Adv. 2022 Apr 19;12(19):11992–5. doi: 10.1039/d2ra01576e (PMC9016497; doi:10.1039/d2ra01576e)

Electronic Supplementary Material (ESI) for RSC Advances.
This journal is © The Royal Society of Chemistry 2022

Supporting information

Direct Sialic Acid 4-OAc substitution by nitrogen, sulfur and carbon nucleophiles with retention of stereochemistry.

Tiago Bozzola, Ulf J. Nilsson and Ulf Ellervik

AUTHOR ADDRESS. Centre for Analysis and Synthesis, Department of Chemistry, Lund University, P.O. Box 124, SE-221 00 Lund, Sweden.

Contents

General Information....................2

General procedure for 4-substitution....................2

Reactions starting from compound 2....................2

Reactions starting from compound 3....................4

^{1}H- and ^{13}C-NMR spectra....................7

General Information

All moisture-and air-sensitive reactions were carried out under an atmosphere of dry nitrogen using oven-dried glassware. All solvents were dried using an MBRAUN SPS-800 Solvent purification system before use unless otherwise stated. Purchased reagents were used without further purification. Thin-layer chromatography was performed on precoated TLC glass plates with silica gel 60 F254 0.25 mm (Merck). Spots were visualized with UV light or by charring with 10% H_2SO_4 in ethanol. Biotage Isolute phase separators were used for drying of combined organic layers. Preparative chromatography was performed on a Biotage Isolera One flash purification system using Biotage SNAP Sfär HD silica cartridges. Optical rotationswere measured on a Bellingham and Stanley model ADP450 polarimeter and are reported as $[\alpha]_D^T$ (c = g/100 mL), where D indicates the sodium D line (589 nm) and T indicates the temperature. NMR spectra were recorded at ambient temperatures on a Bruker Avance II at 400 MHz (^{1}H) and 100 MHz (^{13}C) or a Bruker Ascend at 500 MHz (^{1}H) and 125 MHz (^{13}C) and assigned using 2D methods (COSY, HMQC). Chemical shifts are reported in ppm, with reference to residual solvent peaks (δH $CHCl_3$ = 7.26 ppm, CD_3OH = 3.31 ppm) and solvent signals (δC $CDCl_3$ = 77.0 ppm, CD_3OD = 49.0 ppm). Coupling constant values are given in Hz. Mass spectra were recorded on Waters XEVO G2 (positive ESI). Infrared spectroscopy was recorded on a Bruker *α* II FT-IR spectrometer. IR was only used to confirm structural features, and only the peak of interest was reported.

General procedure for 4-substitution.

Starting material **2** or **3** was dissolved in dry pyridine, the mixture was allowed to attain the correct temperature, and the desired amine (3-10 eq) was added. The reaction was monitored via TLC and LCMS until either full conversion, or appearance of side products. The reaction mixture was then evaporated, and the residues dissolved in EtOAc, washed with distilled water and brine, dried and purified by flash chromatography.

Reactions starting from compound 2

Methyl 5-acetamido-4-benzylamino-7,8,9-tri-O-acetyl-3,4,5-trideoxy-D-glycero-β-D-galacto-non-2-ulopyranosidonate (4a) Reaction according to general procedure: **2** (100 mg, 0.187 mmol), pyridine (2 mL), benzylamine (0.102 mL, 0.937 mmol), -5 °C, 3 days. Flash chromatography (DCM:MeOH 100:0% →96:4 over 4 CV, then 96:4 →95:5 for 6 CV, then isocratic) yielded compound **4a** as an amorphous white solid (25 mg, 25%). $[\alpha]^{20}_D$ -34 (c 1, CH_2Cl_2). ^{1}H NMR (400 MHz, $CDCl_3$) δ 7.39 – 7.24 (m, 5H, Ar-H), 6.06 (d, *J* = 9.1 Hz, 1H, NH), 5.26 (dd, *J* = 5.8, 2.1 Hz, 1H, H-7), 5.21 (ddd, *J* = 7.3, 5.8, 2.3 Hz, 1H, H-8), 4.50 (dd, *J* = 12.3, 2.3 Hz, 1H, H-9), 4.15 (dd, *J* = 10.8, 2.1 Hz, 1H, H-6), 4.13 – 3.93 (m, 3H, H-5, H-9, Ar-CH_2), 3.83 (s, 3H, $COOCH_3$), 3.57 (d, *J* = 12.9 Hz, 1H, Ar-CH_2), 3.00 (q, *J* = 3.5 Hz, 1H, H-4), 2.23 – 1.96 (m, 14H, H-3eq, H-3ax, $OCOCH_3$ x3), 1.79 (s, 3H, $NHCOCH_3$). ^{13}C NMR (101 MHz, $CDCl_3$) δ 170.9, 170.7, 170.5, 170.1, 169.5, 139.8, 128.8, 128.8, 128.6, 127.6, 95.7, 71.4, 68.5, 67.7, 63.1, 53.7, 53.4, 52.5, 46.3, 31.9, 23.4, 21.2, 21.0, 20.9. HRMS (m/z): Calcd. for $C_{25}H_{34}N_2O_{11}$+ [M+H]+, 539.2241; found, 539.2238.

Methyl 5-acetamido-4-(*N*-benzyl-*N*-methyl)amino-7,8,9-tri-O-acetyl-3,4,5-trideoxy-D-glycero-β-D-galacto-non-2-ulopyranosidonate (4b) Reaction according to general procedure: **2** (100 mg, 0.187 mmol), pyridine (2 mL), with *N*-methylbenzylamine (0.241 mL, 1.870 mmol), 7 days. Flash chromatography (DCM:MeOH 100:0% →96:4 over 4 CV, then 96:4 →95:5 for 6 CV, then isocratic) yielded compound **4b** as an amorphous white solid (40 mg, 39%). $[\alpha]^{20}_D$ +4 (c 1, CH_2Cl_2).

^{1}H NMR (400 MHz, $CDCl_3$) δ 7.34 – 7.19 (m, 6H, Ar-H), 5.30 (dd, *J* = 6.2, 2.2 Hz, 1H, H-7), 5.20 (ddd, *J* = 7.3, 6.2, 2.4 Hz, 1H, H-8), 4.96 (d, *J* = 9.9 Hz, 1H, NH), 4.44 (dd, *J* = 12.3, 2.4 Hz, 1H, H-8), 4.17 (q, *J* = 10.4 Hz, 1H, H-5), 4.04 – 3.95 (m, 2H, H-6. H-9), 3.88 (s, 3H, $COOCH_3$), 3.69 (d, *J* = 13.3 Hz, 1H, Ar-CH_2), 3.44 (d, *J* = 13.3 Hz, 1H, Ar-CH_2), 3.03 (td, *J* = 11.6, 4.0 Hz, 1H, H-4), 2.22 (s, 3H), 2.14 (s, 4H), 2.10 – 1.90 (m, 11H, H-3_{eq}, H-3_{ax}, CH_3N, $HNCOCH_3$, $OCOCH_3$ x 3). ^{13}C NMR (101 MHz, $CDCl_3$) δ 170.9, 170.6, 170.5, 170.4, 170.1, 139.8, 128.6, 128.4, 127.2, 95.4, 72.4, 71.1, 68.6, 63.0, 59.4, 57.9, 53.6, 47.1, 36.9, 29.0, 23.6, 21.2, 21.1, 20.9. HRMS (m/z): Calcd. for $C_{26}H_{36}N_2O_{11}$+ [M+H]+, 553.2397; found, 553.2403.

Methyl 5-acetamido-4-phenylamino-7,8,9-tri-O-acetyl-3,4,5-trideoxy-D-glycero-β-D-galacto-non-2-ulopyranosidonate (4c) Reaction according to general procedure: **2** (100 mg, 0.187 mmol), pyridine (2 mL), aniline (0.170 mL, 1.870 mmol), 30 days. Flash chromatography (DCM:MeOH 100:0% →96:4 over 4 CV, then 96:4 →95:5 for 6 CV, then isocratic) yielded compound **4c** as an amorphous white solid (33 mg, 34%). $[\alpha]^{20}_D$ +14 (c 1, CH_2Cl_2).^{1}H NMR (400 MHz, $CDCl_3$) δ 7.20 – 7.07 (m, 2H, Ar-H), 6.72 – 6.52 (m, 3H, Ar-H), 5.63 (d, *J* = 10.1 Hz, 1H, AcNH), 5.41 (dd, *J* = 5.2, 2.4 Hz, 1H, H-7), 5.28 (ddd, *J* = 7.4, 5.1, 2.3 Hz, 1H, H-8), 4.54 (dd, *J* = 12.4, 2.3 Hz, 1H, H-9), 4.28 (dd, *J* = 10.4, 2.4 Hz, 1H, H-6), 4.12 – 3.96 (m, 2H, H-5, H-9), 3.87 (d, *J* = 9.7 Hz, 1H, Ar-NH), 3.85 (s, 3H, $COOCH_3$), 3.80 (td, *J* = 11.2, 4.3 Hz, 1H, H-4), 2.39 (dd, *J* = 13.5, 4.2 Hz, 1H, H-3_{eq}), 2.20 – 2.01 (m, 9H, $OCOCH_3$ x 3), 1.96 (dd, *J* = 13.4, 11.6 Hz, 1H, H-3_{ax}), 1.74 (s, 3H, $HNCOCH_3$). ^{13}C NMR (101 MHz, $CDCl_3$) δ 171.8, 171.1, 171.0, 170.3, 169.8, 147.4, 129.5, 117.8, 113.2, 95.1, 71.8, 71.7, 68.6, 62.8, 53.6, 52.4, 50.3, 37.9, 23.2, 21.2, 20.9, 20.8. HRMS (m/z): Calcd. for $C_{24}H_{32}N_2O_{11}$+ [M+H]+, 525.2084; found, 525.2081.

Methyl 5-acetamido-4-indolin-1-yl-7,8,9-tri-O-acetyl-3,4,5-trideoxy-D-glycero-β-D-galacto-non-2-ulopyranosidonate (4d) Reaction according to general procedure: **2** (100 mg, 0.187 mmol), pyridine (2 mL), indoline (0.133 mL, 1.870 mmol), 30 days. Flash chromatography (Hep:EtOAc 90:10→40:60 over 5 CV, then 40:60→20:80 for 10 CV then isocratic yielded compound **4d** as an amorphous white solid (68 mg, 66%). $[\alpha]^{20}_D$ -3 (c 1, CH_2Cl_2). ^{1}H NMR (400 MHz, $CDCl_3$) δ 7.06 – 6.92 (m, 2H, Ar-H), 6.58 (td, *J* = 7.4, 1.0 Hz, 1H, Ar-H), 6.41 (d, *J* = 7.7 Hz, 1H, Ar-H), 5.37 (dd, *J* = 5.7, 2.3 Hz, 1H, H-7), 5.28 (ddd, *J* = 7.4, 5.7, 2.4 Hz, 1H, H-8), 5.22 (d, *J* = 10.0 Hz, 1H, NH), 4.51 (dd, *J* = 12.3, 2.4 Hz, 1H, H-9), 4.38 – 4.29 (m, 1H, H-5), 4.25 (dd, *J* = 10.3, 2.3 Hz, 1H, H-6), 4.08 – 3.95 (m, 2H, H-4, H-9), 3.86 (s, 3H, $COOCH_3$), 3.56 (td, *J* = 8.9, 5.9 Hz, 1H, indoline), 3.29 (q, *J* = 9.0 Hz, 1H, indoline), 3.02 – 2.83 (m, 2H, indoline), 2.30 – 2.21 (m, 1H, H-3eq), 2.15 (s, 3H, $OCOCH_3$), 2.11 (s, 3H, $OCOCH_3$), 2.03 (s, 3H, $OCOCH_3$), 1.97 (dd, *J* = 13.1, 4.1 Hz, 1H, H-3ax), 1.66 (s, 3H, $HNCOCH_3$). ^{13}C NMR (101 MHz, $CDCl_3$) δ 170.96, 170.86, 170.51, 170.47, 169.79, 151.18, 130.04, 127.29, 125.06, 117.59, 106.15, 95.18, 72.37, 71.42, 68.44, 62.97, 53.63, 52.58, 47.28, 46.12, 32.30, 28.25, 23.21, 21.24, 21.05, 20.99. HRMS (m/z): Calcd. for $C_{26}H_{34}N_2O_{11}$+ [M+H]+, 551.2241; found, 551.2241.

Methyl 5-acetamido-4-tosylamino-7,8,9-tri-O-acetyl-3,4,5-trideoxy-D-glycero-β-D-galacto-non-2-ulopyranosidonate (4e) p-toluenesulfonamide (320 mg, 1.870 mmol) was dissolved in dry pyridine and potassium tert-butoxide (68 mg, 0.561 mmol) then added. After 10 minutes, starting material **2** (100 mg, 0.187) was subsequently added. The reaction was stopped after 3 days, concentrated, washed with water and brine, dried and flash chromatography performed (Hep:EtOAc 75:25→10:90 over 4 CV, then isocratic). The reaction yielded compound **4e** as an amorphous white solid (23 mg, 20%). $[\alpha]^{20}_D$ +18 (c 1, CH_2Cl_2). ^{1}H NMR (400 MHz, $CDCl_3$) δ 7.71 (d, *J* = 8.3 Hz, 2H, Ar-H), 7.29 (d, *J* = 7.9 Hz, 2H, Ar-H), 5.41 (d, *J* = 9.9 Hz, 1H, AcNH), 5.31 (dd, *J* = 6.2,

2.2 Hz, 1H, H-7), 5.19 (td, *J* = 6.8, 2.5 Hz, 2H, H-8, S-NH), 4.40 (dd, *J* = 12.4, 2.3 Hz, 1H, H-8), 4.19 – 4.09 (m, 1H, H-6), 4.00 (dd, *J* = 12.4, 6.9 Hz, 1H, H-9), 3.88 (q, *J* = 10.3 Hz, 1H, H-5), 3.83 (s, 3H, $COOCH_3$), 3.69 – 3.55 (m, 1H, H-4), 2.42 (s, 3H, Ar-CH_3), 2.19 – 1.97 (m, 11H, H-3_{eq}, H-3_{ax}, $OCOCH_3$ x 3), 1.81 (s, 3H, $HNCOCH_3$). ^{13}C NMR (101 MHz, $CDCl_3$) δ 174.6, 172.2, 170.8, 170.5, 170.1, 143.6, 138.3, 129.9, 126.9, 94.5, 71.1, 70.7, 68.1, 62.7, 60.5, 53.7, 49.2, 38.6, 23.3, 21.7, 21.1, 20.9, 20.8. ESI-MS (m/z): Calcd. for $C_{25}H_{34}N_2O_{13}S^+$ [M+H]+, 603.1860; found, 603.1854.

Reactions starting from compound 3

Methyl 5-acetamido-4-benzylamino-7,8,9-tri-O-acetyl-3,4,5-trideoxy-D-glycero-β-D-galacto-non-2-ulopyranosidonate (4a) Reaction according to general procedure: **3** (100 mg, 0.203 mmol), pyridine (2 mL), benzylamine (0.044 mL, 0.407 mmol), -10°C, 20 hours. Flash chromatography conditions (DCM:MeOH 100:0% →96:4 over 4 CV, then 96:4 →95:5 for 6 CV, then isocratic) yielded compound **4a** as an amorphous white solid (37 mg, 34%). Physical data as reported starting from compound **2**.

Methyl 5-acetamido-4-phenylamino-7,8,9-tri-O-acetyl-3,4,5-trideoxy-D-glycero-β-D-galacto-non-2-ulopyranosidonate (4c) Reaction according to general procedure: **3** (100 mg, 0.203 mmol), pyridine (2 mL), aniline (0.185 mL, 2.304 mmol). 17 days. Flash chromatography conditions (DCM:MeOH 100:0% →96:4 over 4 CV, then 96:4 →95:5 for 6 CV, then isocratic) yielded compound **4c** as an amorphous white solid (42 mg, 39%). Physical data as reported starting from compound **2**.

Methyl 5-acetamido-4-tosylamino-7,8,9-tri-O-acetyl-3,4,5-trideoxy-D-glycero-β-D-galacto-non-2-ulopyranosidonate (4e) p-toluenesulfonamide (174 mg, 1.017 mmol) was dissolved in dry pyridine and caesium carbonate (133 mg, 0.407 mmol) then added. After 10 minutes, starting material **3** (100 mg, 0.203) was subsequently added. The reaction was stopped after 2 hours, concentrated, washed with water and brine, dried and flash chromatography performed (Hep:EtOAc 75:25→10:90 over 4 CV, then isocratic). The reaction yielded compound **4e** as an amorphous white solid (31 mg, 25%). Physical data as reported starting from compound **2**.

Methyl 5-acetamido-4-azido-7,8,9-tri-O-acetyl-3,4,5-trideoxy-D-glycero-β-D-galacto-non-2-ulopyranosidonate (4f) Starting material **3** (1.0 g, 2.03 mmol) was reacted with sodium azide (0.396 g, 6.09 mmol). The reaction was completed after 6 days and the reaction mixture filtered, evaporated and flash chromatography performed directly (Hep:EtOAc 60:40 → 20:80 over 6 CV, then isocratic). The purification afforded **4f** as an amorphous white solid (438 mg, 45%). $[\alpha]^{20}_D$ +18 (c 0.5, CH_2Cl_2). ^{1}H NMR (500 MHz, $CDCl_3$) δ 5.52 (d, *J* = 9.4 Hz, 1H, AcN-H), 5.32 (dd, *J* = 6.5, 2.0 Hz, 1H, H-7), 5.24 (td, *J* = 6.6, 2.4 Hz, 1H, H-8), 4.41 (dd, *J* = 12.4, 2.3 Hz, 1H, H-9), 4.30 (dd, *J* = 10.5, 2.0 Hz, 1H, H-6), 4.05 (m, 2H, H-9, H-4), 3.87 (s, 3H), 3.80 – 3.66 (m, 1H, H-5), 2.21 (dd, *J* = 13.1, 4.9 Hz, 1H, H-3_{eq}), 2.15 – 1.99 (m, 13H, H-3_{ax}, $OCOCH_3$ x 3, $HNCOCH_3$). ^{13}C NMR (126 MHz, $CDCl_3$) δ 171.3, 170.9, 170.6, 169.2, 94.5, 70.5, 70.0, 68.0, 62.7, 58.0, 53.8, 51.1, 36.2, 23.6, 21.2, 21.1, 21.0, 20.9. ESI-MS (m/z): Calcd. for $C_{18}H_{26}N_4O_{11}$ [M+Na]+, 497.1496; found, 497.1490. IR: 2106 cm^{-1} (N_3).

Methyl 5-acetamido-4-(*N*,*N*-diethylamino) -7,8,9-tri-O-acetyl-3,4,5-trideoxy-D-glycero-β-D-galacto-non-2-ulopyranosidonate (4g) Starting material **3** (100 mg, 0.203 mmol) was reacted with diethylamine (0.105 mL, 1.015 mmol). The reaction was completed after 4 hours and the reaction mixture evaporated and flash chromatography performed directly (DCM-MeOH 100:0 → 20:80

over 10 CV, then isocratic). The purification afforded **4g** as an amorphous white solid (42 mg, 41%). $[\alpha]^{20}_{D}$ -12 (c 1, CH_2Cl_2). ^{1}H NMR (500 MHz, $CDCl_3$) δ 7.45 (d, *J* = 10.3 Hz, 1H, AcN-H), 5.30 (m, 1H, H-8), 5.26 (dd, *J* = 8.1, 1.7 Hz, H-7), 4.44 (q, *J* = 10.4 Hz, 1H, H-5), 4.29 (dd, *J* = 12.3, 2.5 Hz, 1H, H-9), 4.14 (m, 2H, H-4, H-6), 4.01 (dd, *J* = 12.4, 6.9 Hz, 1H, H-9), 3.90 (s, 3H, $COOCH_3$), 3.61 – 3.51 (m, 1H, N-CH_2-), 3.50 – 3.41 (m, 1H, N-CH_2-), 2.89 – 2.79 (m, 1H, N-CH_2-), 2.75 – 2.64 (m, 1H, N-CH_2-), 2.24 (t, *J* = 12.5 Hz, 1H, H-3_{eq}), 2.20 – 2.00 (m, 13H, , H-3_{ax}, $OCOCH_3$ x 3, $HNCOCH_3$), 1.58 (t, *J* = 7.1 Hz, 3H, NCH_2CH_3), 1.40 (t, *J* = 7.2 Hz, 3H NCH_2CH_3). ^{13}C NMR (126 MHz, $CDCl_3$) δ 172.45, 170.84, 170.18, 168.34, 94.46, 72.11, 69.72, 67.24, 62.69, 57.70, 53.84, 47.57, 46.53, 44.89, 30.11, 23.74, 21.30, 21.09, 20.93, 10.72, 9.84. ESI-MS (m/z): Calcd. for $C_{22}H_{36}N_2O_{11}$+ [M+H]+, 505.2397; found, 505.2404.

Methyl 5-acetamido-4-cyano-7,8,9-tri-O-acetyl-3,4,5-trideoxy-D-glycero-β-D-galacto-non-2-ulopyranosidonate (4h) Starting material **3** (100 mg, 0.203 mmol) was reacted with potassium cyanide (66 mg, 1.015 mmol). The reaction was completed after 2 days and the reaction mixture evaporated and flash chromatography performed directly (Hep:EtOAc 60:40 → 20:80 over 6 CV, then isocratic). The purification afforded **4h** as an amorphous white solid (35 mg, 38%). $[\alpha]^{20}_{D}$ +7 (c 1, CH_2Cl_2). ^{1}H NMR (500 MHz, $CDCl_3$) δ 5.79 (d, *J* = 7.4 Hz, 1H, AcN-H), 5.35 – 5.19 (m, 2H, H-7, H-8), 4.43 (d, *J* = 9.5 Hz, 1H, H-6), 4.38 (dd, *J* = 11.6, 1.8 Hz, 1H, H-9), 4.08 (dd, *J* = 11.6, 4.5 Hz, 1H, H-9), 3.88 (s, 3H. $COOCH_3$), 3.74 – 3.66 (m, 2H, H-4, H-5), 2.42 (t, *J* = 13.0 Hz, 1H, H-3_{eq}), 2.25 (dd, *J* = 13.0, 3.4 Hz, 1H, H-3_{ax}), 2.20 – 1.98 (m, 14H, $OCOCH_3$ x 3, $HNCOCH_3$). ^{13}C NMR (126 MHz, $CDCl_3$) δ 170.8, 170.8, 170.6, 170.6, 168.8, 119.1, 93.1, 70.0, 69.2, 68.1, 62.6, 53.8, 48.6, 34.1, 28.4, 23.6, 21.2, 21.1, 21.0, 20.9, 14.3. ESI-MS (m/z): Calcd. for $C_{19}H_{26}N_2O_{11}$+ [M+Na]+, 481.1434; found, 481.1433.

Methyl 5-acetamido-4-(p-tolylthio)-7,8,9-tri-O-acetyl-3,4,5-trideoxy-D-glycero-β-D-galacto-non-2-ulopyranosidonate (4i) Reaction according to general procedure: **3** (50 mg, 0.102 mmol), pyridine (2 mL), thiocresol (63 mg, 0.509 mmol), Cs_2CO_3 (66 mg, 0.203 mmol). 4 hours. Flash chromatography conditions (DCM:MeOH 100:0% →96:4 over 4 CV, then 96:4 →95:5 for 6 CV, then isocratic) yielded compound **4i** as an amorphous white solid (51 mg, 89%). $[\alpha]^{20}_{D}$ +20 (c 1, CH_2Cl_2). ^{1}H NMR (500 MHz, $CDCl_3$) δ 7.33 (d, *J* = 7.9 Hz, 2H, Ar-H), 7.10 (d, *J* = 7.9 Hz, 2H, Ar-H), 5.64 (m, 1H, AcN-H), 5.29 (dd, *J* = 6.2, 2.0 Hz, 1H, H-7), 5.21 (td, *J* = 6.5, 2.3 Hz, 1H, H-8), 4.42 (dd, *J* = 12.4, 2.4 Hz, 1H, H-9), 4.24 (dd, *J* = 10.3, 2.0 Hz, 1H, H-6), 4.01 (dd, *J* = 12.3, 7.2 Hz, 1H, H-9), 3.91 – 3.74 (m, 5H, H-5, $COOCH_3$, 2-OH), 3.59 (td, *J* = 11.7, 4.5 Hz, 1H, H-4), 2.32 (s, 3H, CH3-Ar), 2.23 – 2.00 (m, 8H, H-3_{eq}, H-3_{ax}, $OCOCH_3$ x 3), 1.87 (s, 3H, $HNCOCH_3$). ^{13}C NMR (126 MHz, $CDCl_3$) δ 170.9, 170.7, 170.6, 169.7, 138.1, 133.7, 130.0, 129.2, 94.5, 71.4, 70.9, 68.5, 62.9, 53.6, 50.7, 45.8, 37.8, 23.2, 21.2, 21.2, 21.1, 21.0, 20.9. ESI-MS (m/z): Calcd. for $C_{25}H_{33}NO_{11}S$+ [M+H]+, 556.1853; found, 556.1858.

Methyl 5-acetamido-4-(benzylthio)-7,8,9-tri-O-acetyl-3,4,5-trideoxy-D-glycero-β-D-galacto-non-2-ulopyranosidonate (4l) Reaction according to general procedure: **3** (35 mg, 0.0712 mmol), pyridine (2 mL), benzyl mercaptan (0.040 mL, 0.356 mmol), Cs_2CO_3 (46 mg, 0.142 mmol). 4 hours. Flash chromatography conditions (DCM:MeOH 100:0% →96:4 over 4 CV, then 96:4 →95:5 for 6 CV, then isocratic) yielded compound **4l** as an amorphous white solid (25 mg, 63%). $[\alpha]^{20}_{D}$ +33 (c 1, CH_2Cl_2). ^{1}H NMR (500 MHz, $CDCl_3$) δ 7.40 – 7.25 (m, 5H), 5.30 (dd, *J* = 6.3, 2.0 Hz, 2H, H, 7AcN-H), 5.20 (td, *J* = 6.7, 2.3 Hz, 1H, H-8,), 4.42 (dd, *J* = 12.4, 2.4 Hz, 1H, H-9), 4.15 (d, *J* = 9.9 Hz, 1H, H-6), 4.01 (dd, *J* = 12.3, 7.0 Hz, 1H, H-9), 3.94 – 3.83 (m, 4H, H-5, $COOCH_3$),

3.78 (d, J = 12.6 Hz, 1H, Ar-CH_2-S), 3.70 (d, J = 12.6 Hz, 1H, Ar-CH_2-S), 3.06 (td, J = 10.9, 3.0 Hz, 1H, H-4) 2.33 (t, J = 13.0 Hz, 1H, H-3_{eq}), 2.23 (dd, J = 13.4, 4.3 Hz, 1H, H-3_{ax}), 2.17 – 1.91 (m, 12H, $OCOCH_3$ x 3, $HNCOCH_3$). ^{13}C NMR (126 MHz, $CDCl_3$) δ 170.9, 170.6, 170.5, 169.7, 138.1, 129.2, 128.8, 127.4, 94.4, 71.7, 70.8, 68.5, 62.9, 53.6, 48.9, 42.8, 38.4, 34.3, 23.3, 21.2, 21.0, 20.9. ESI-MS (m/z): Calcd. for $C_{25}H_{33}NO_{11}S^+$ [M+Na]+, 556.1853; found, 556.1859.

^{1}H- and ^{13}C-NMR spectra of compound 4a

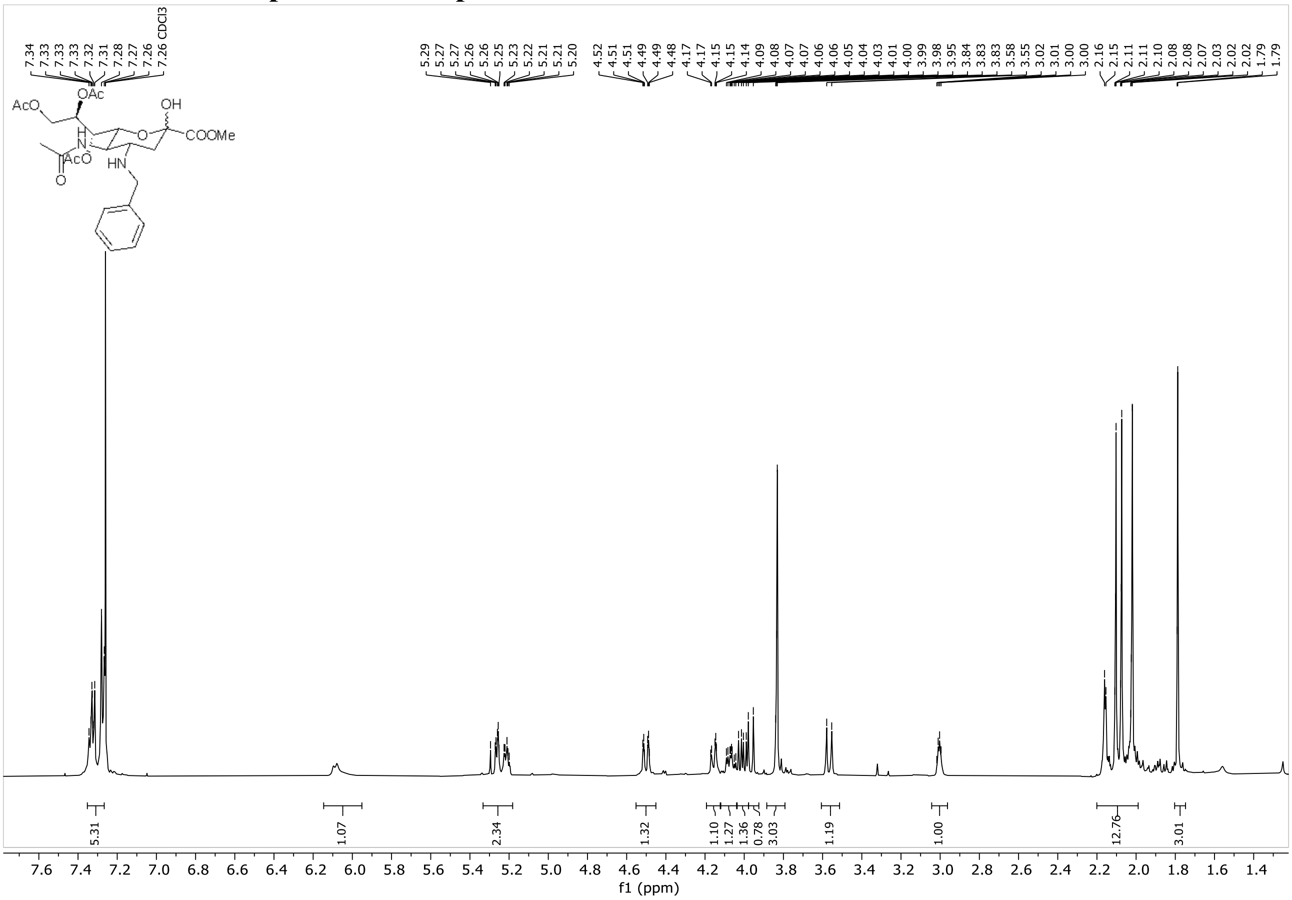

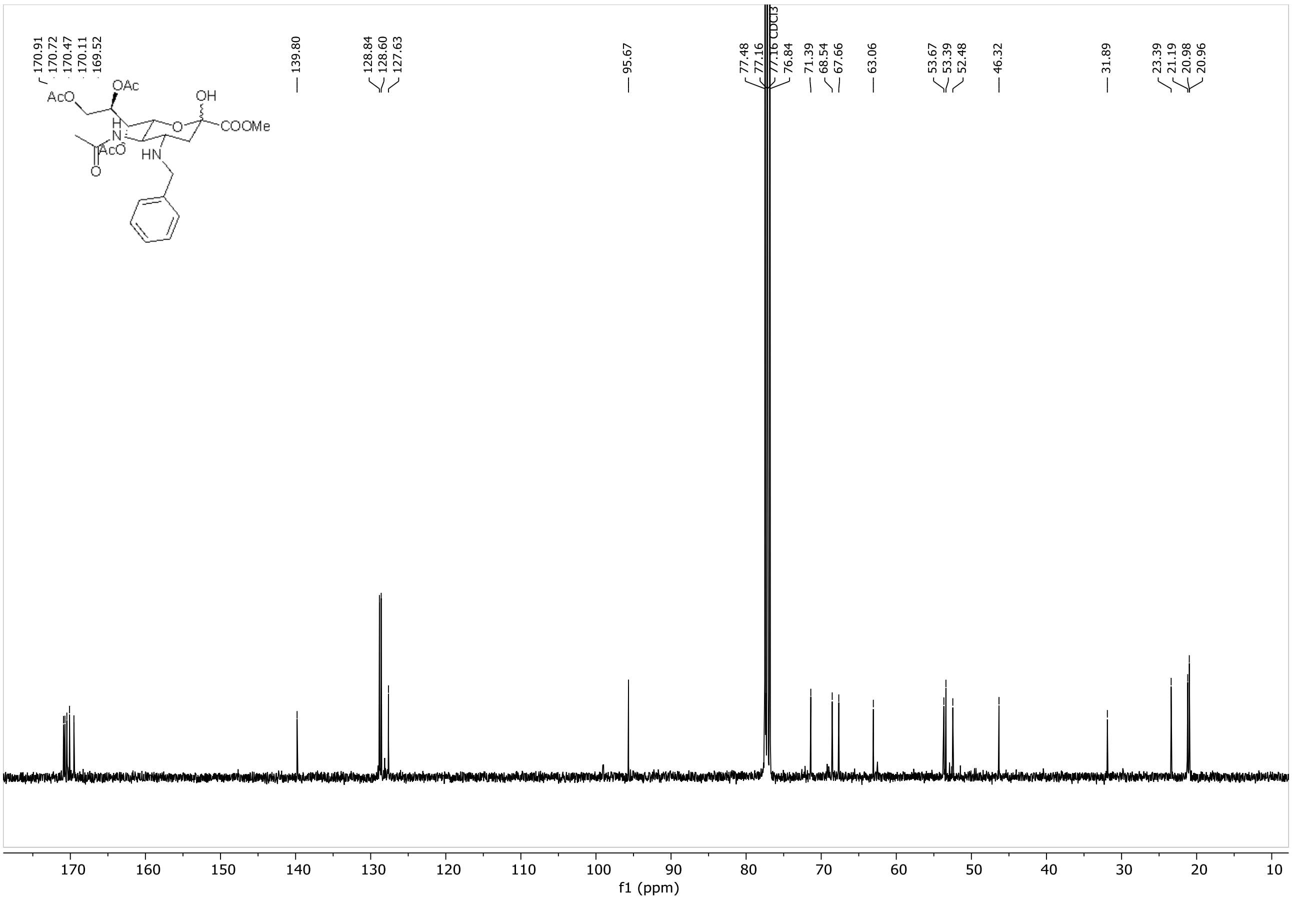

^{1}H- and ^{13}C-NMR spectra of compound 4b

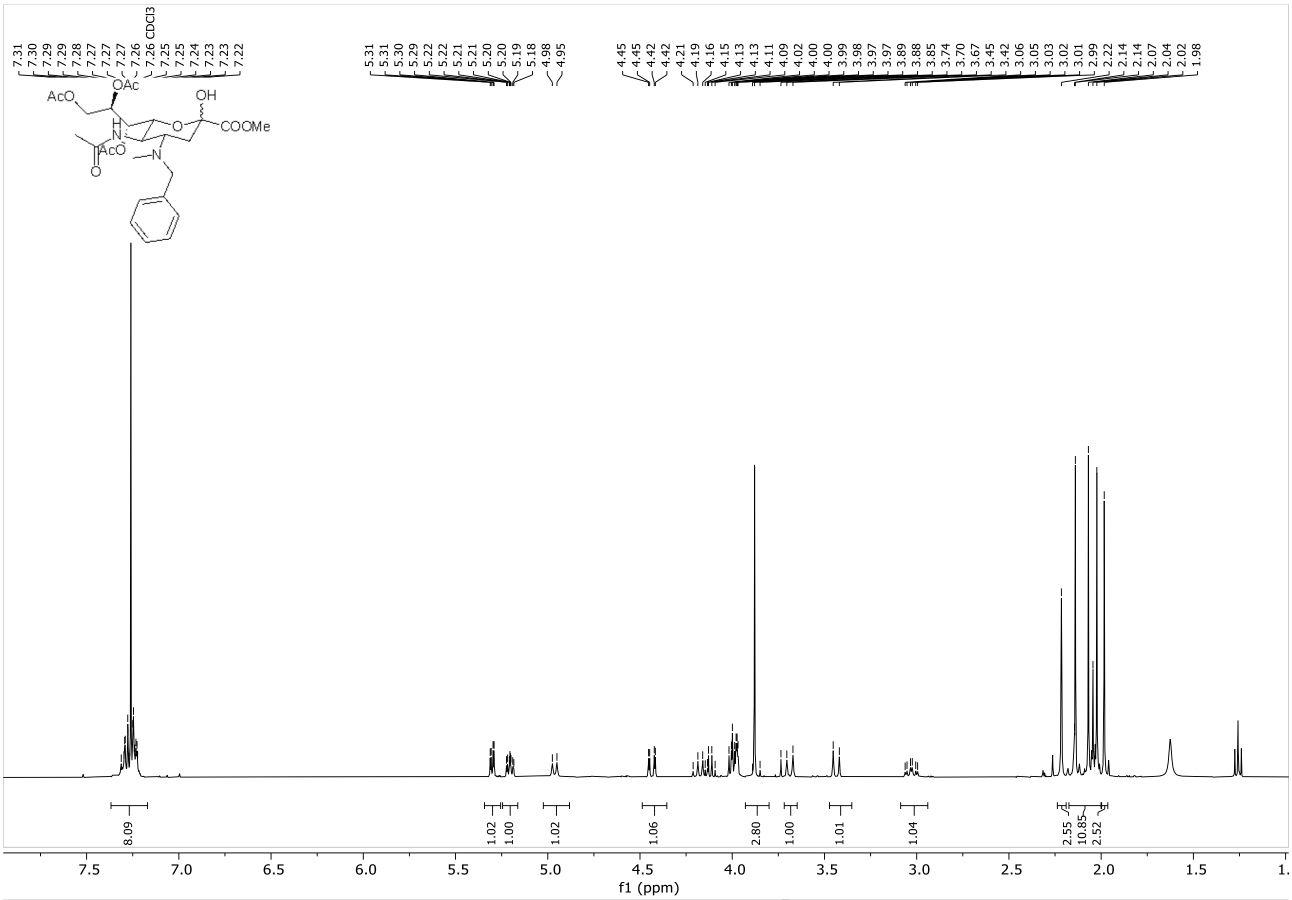

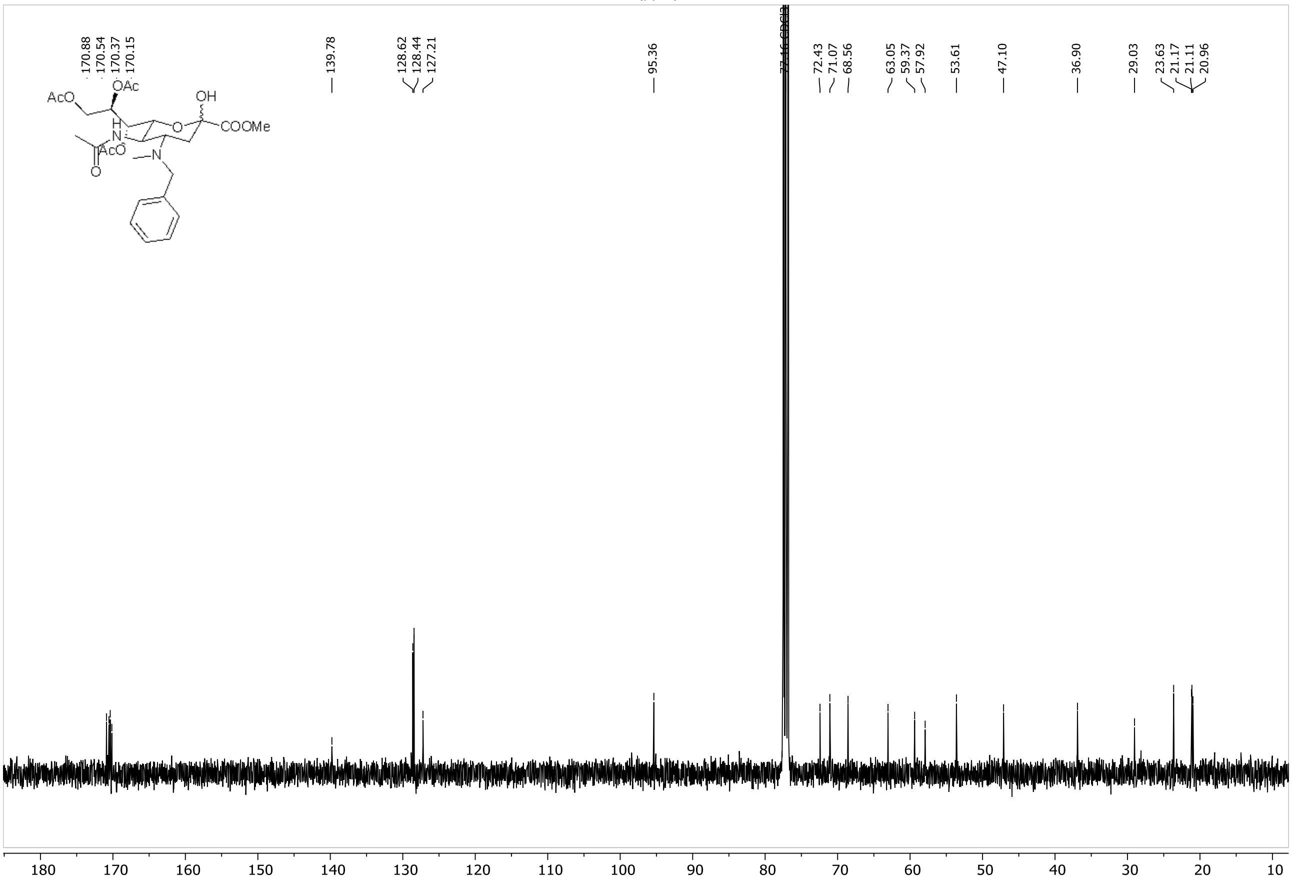

^{1}H- and ^{13}C-NMR spectra of compound 4c

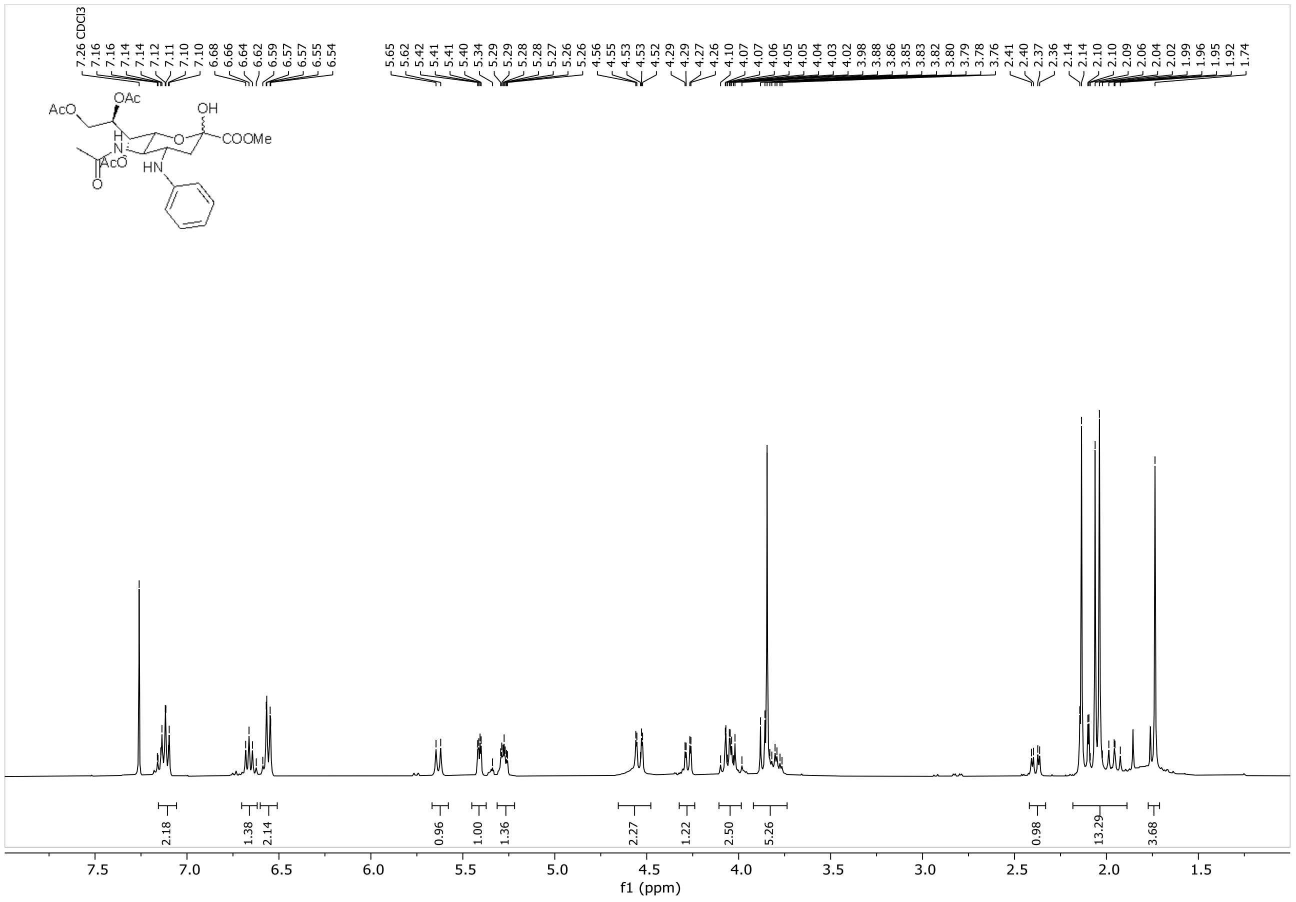

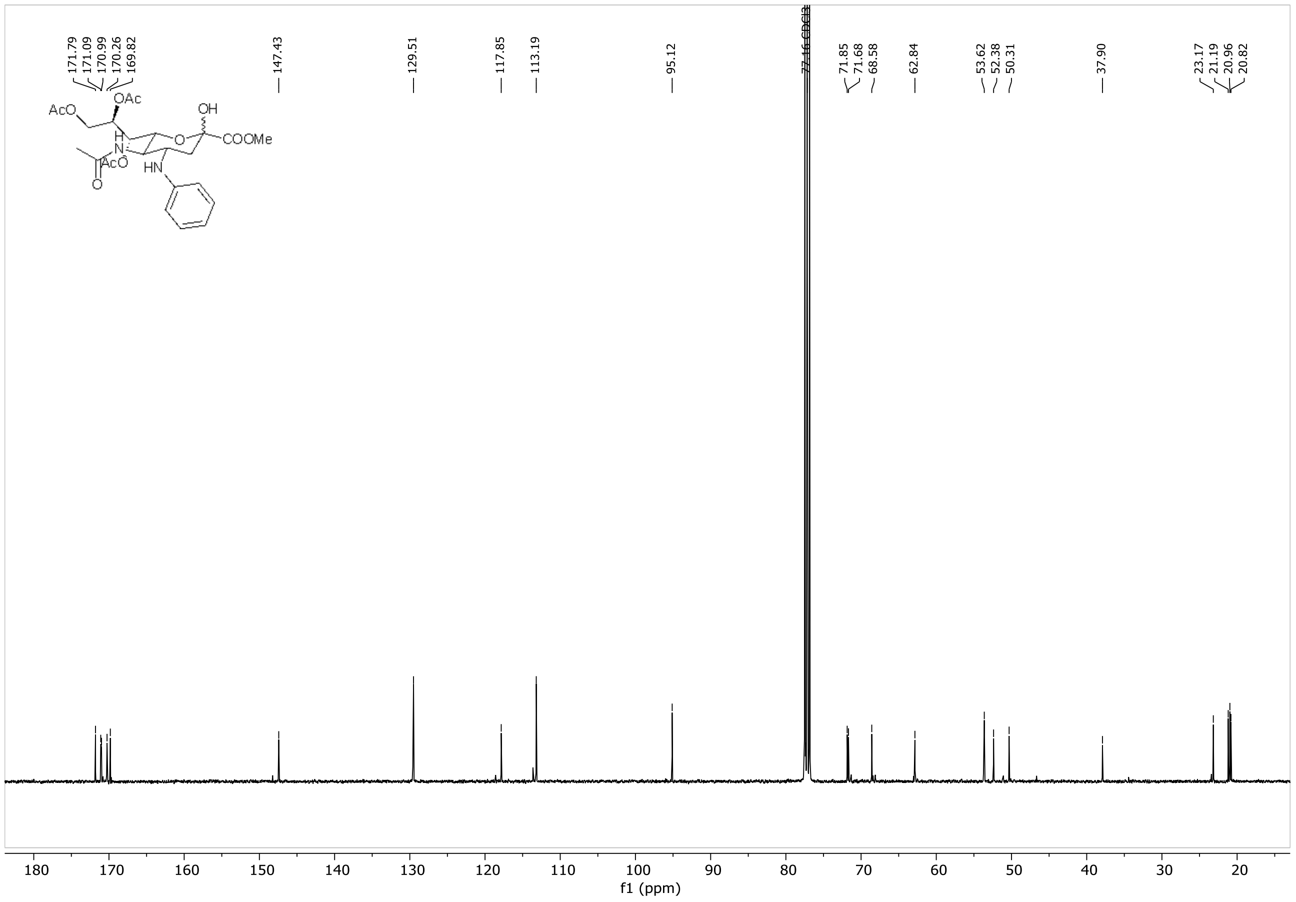

^{1}H- and ^{13}C-NMR spectra of compound 4d

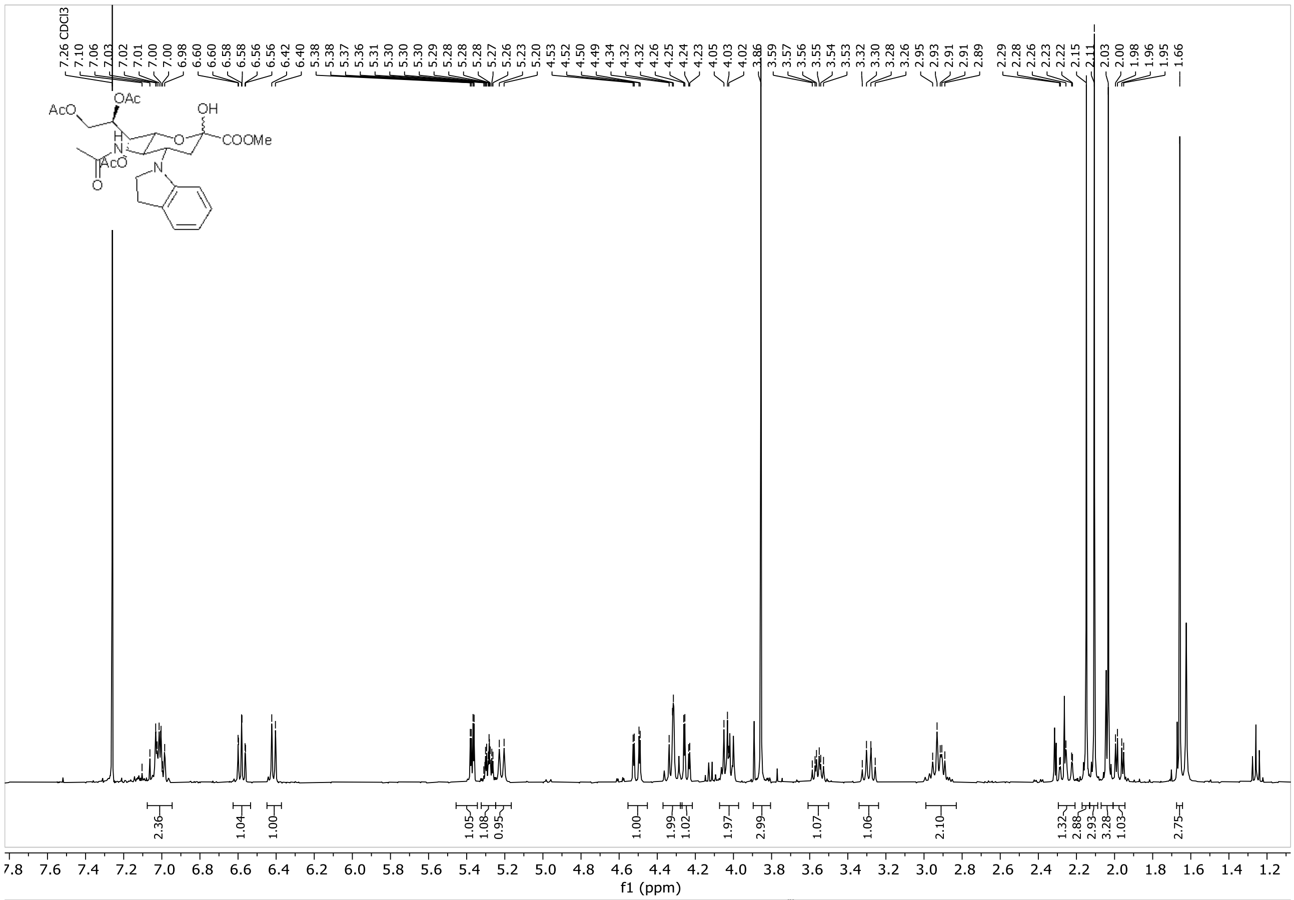
7.26 CDCl3
7.10
7.06
7.03
7.02
7.01
7.00
7.00
6.98
6.60
6.60
6.58
6.58
6.56
6.56
6.42
6.40
5.38
5.38
5.37
5.36
5.31
5.30
5.30
5.30
5.29
5.28
5.28
5.28
5.27
5.26
5.23
5.20
4.53
4.52
4.50
4.49
4.34
4.32
4.32
4.26
4.25
4.24
4.23
4.05
4.02
3.86
3.59
3.57
3.56
3.55
3.54
3.53
3.32
3.30
3.28
3.26
2.95
2.93
2.91
2.91
2.89
2.29
2.28
2.26
2.23
2.22
2.15
2.11
2.03
2.00
1.98
1.96
1.95
1.66
AcO
OAc
OH
H
N
O
COOMe
AcO
O
N
2.36
1.04
1.00
1.05
1.08
0.95
1.00
1.99
1.02
1.97
2.99
1.07
1.06
2.10
1.32
2.88
2.93
3.28
1.03
2.75
7.8 7.6 7.4 7.2 7.0 6.8 6.6 6.4 6.2 6.0 5.8 5.6 5.4 5.2 5.0 4.8 4.6 4.4 4.2 4.0 3.8 3.6 3.4 3.2 3.0 2.8 2.6 2.4 2.2 2.0 1.8 1.6 1.4 1.2
f1 (ppm)

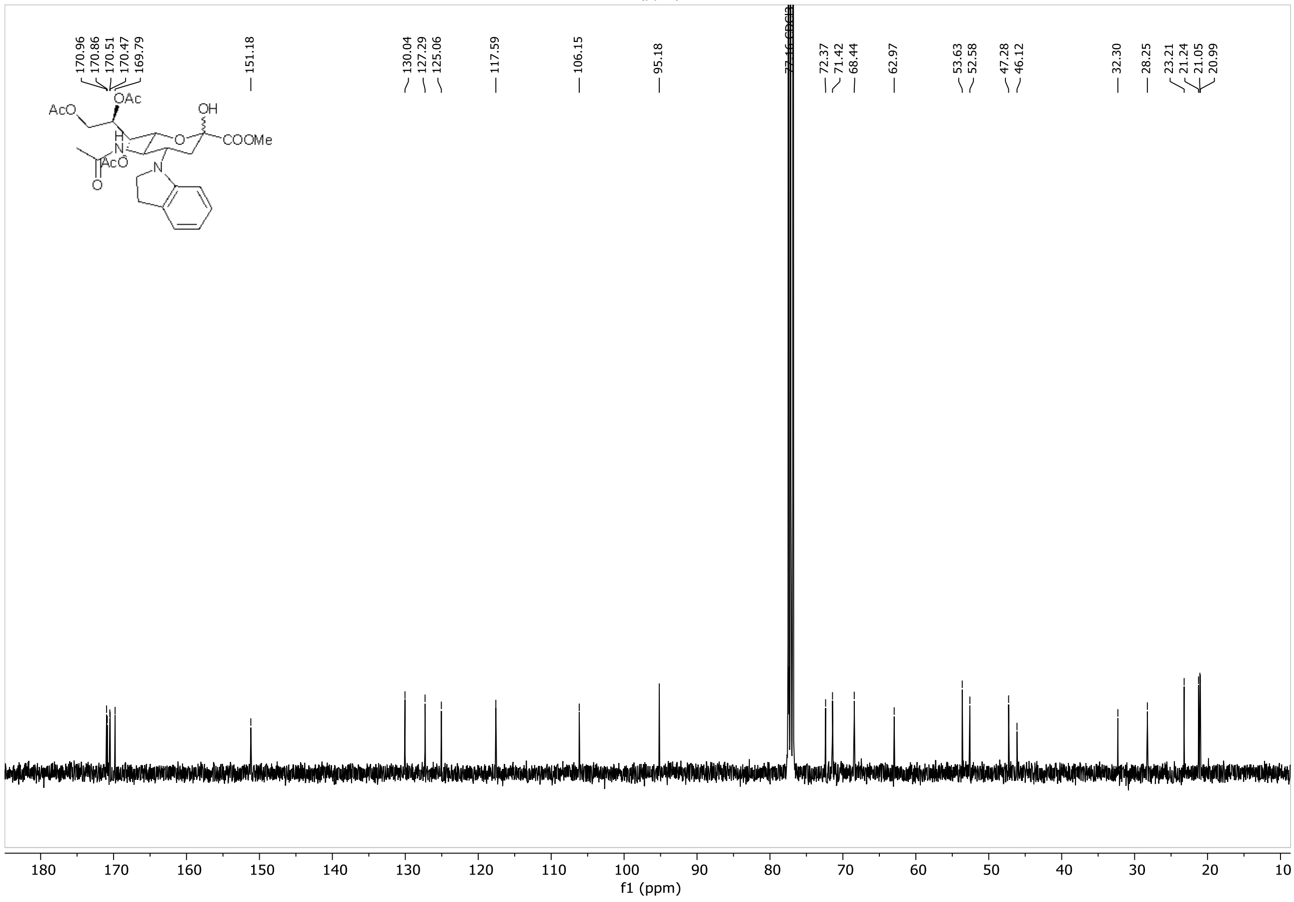
170.96
170.86
170.51
170.47
169.79
151.18
130.04
127.29
125.06
117.59
106.15
95.18
72.37
71.42
68.44
62.97
53.63
52.58
47.28
46.12
32.30
28.25
23.21
21.24
21.05
20.99
AcO
OAc
OH
H
N
O
COOMe
AcO
O
N
180 170 160 150 140 130 120 110 100 90 80 70 60 50 40 30 20 10
f1 (ppm)

^{1}H- and ^{13}C-NMR spectra of compound 4e

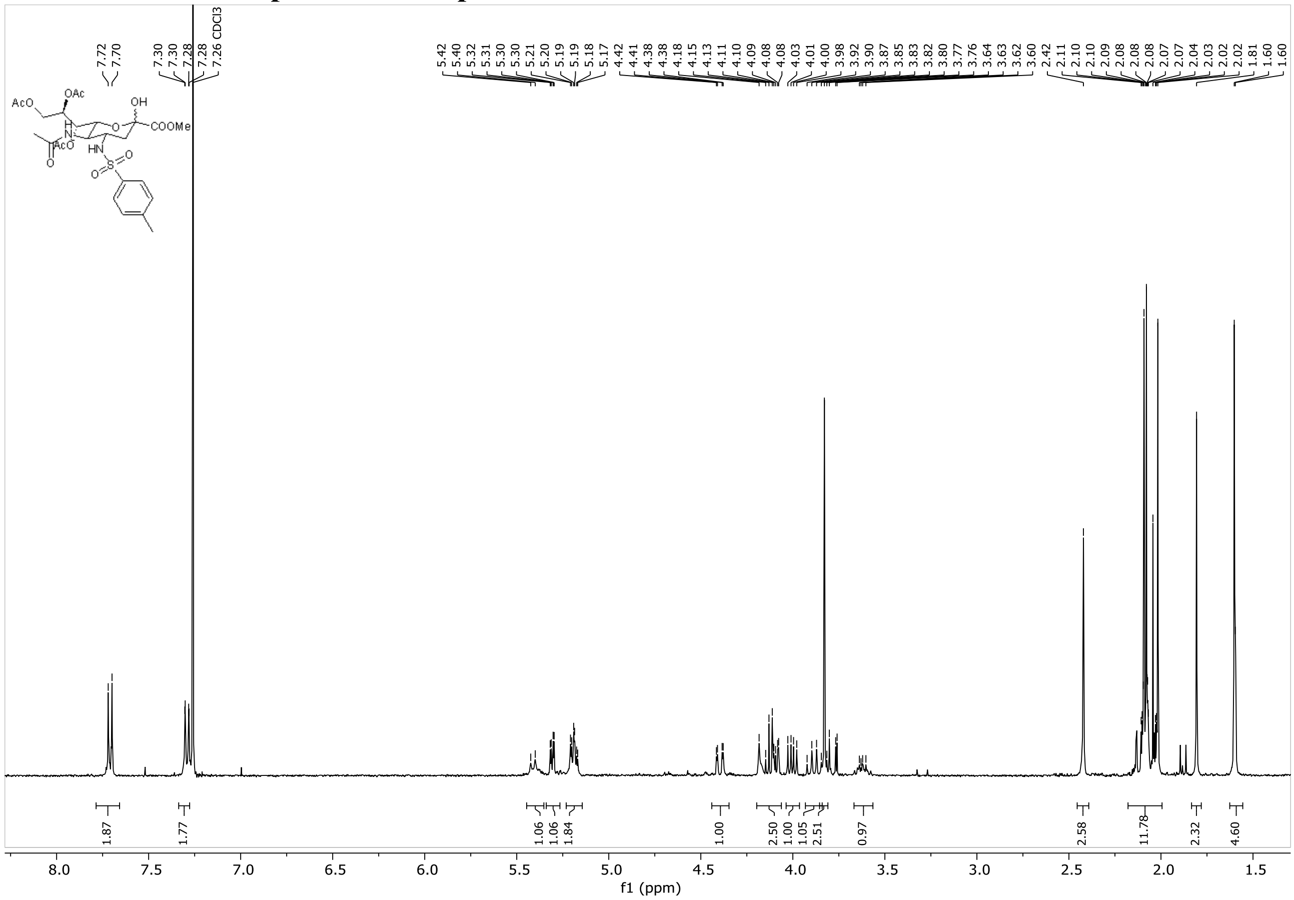

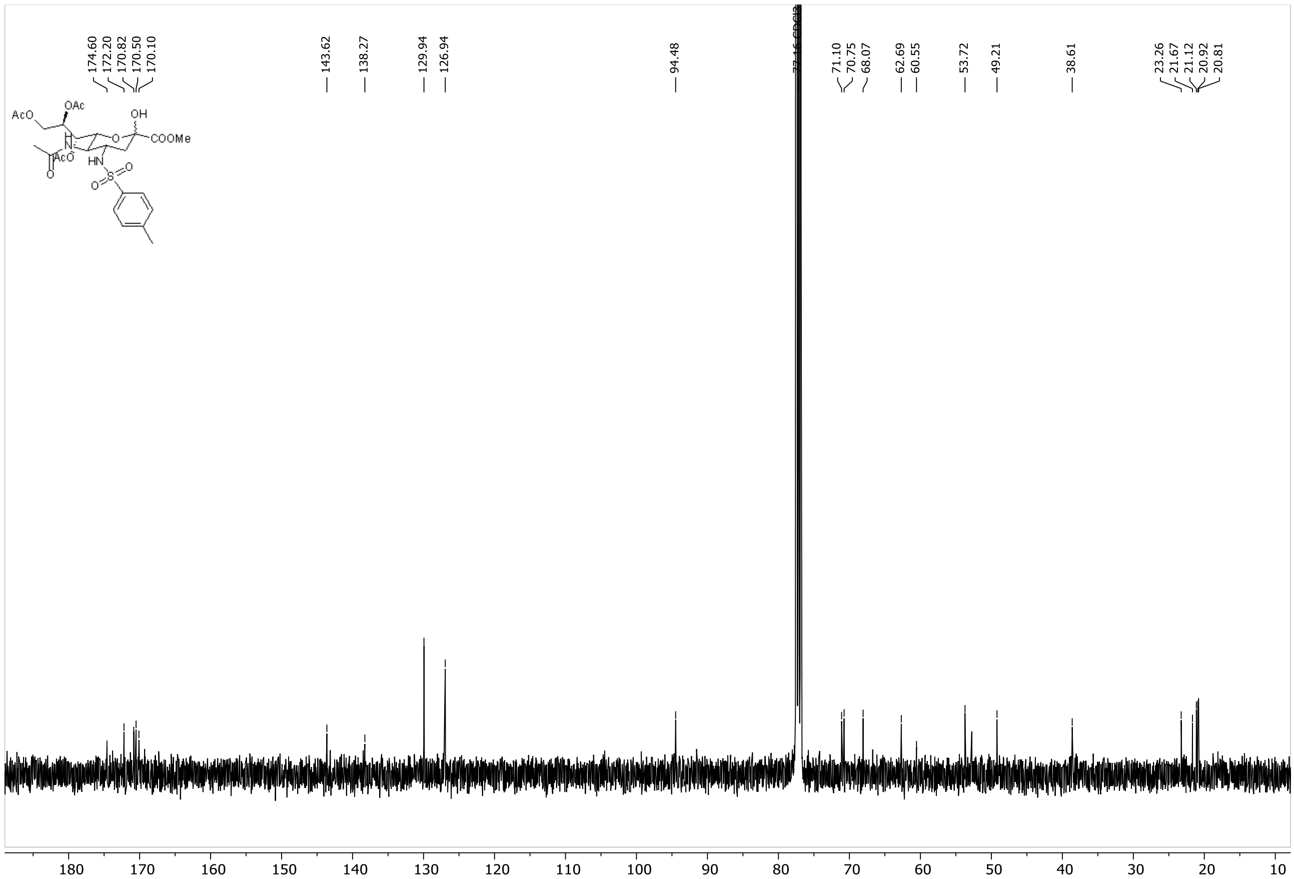

^{1}H- and ^{13}C-NMR spectra of compound 4f

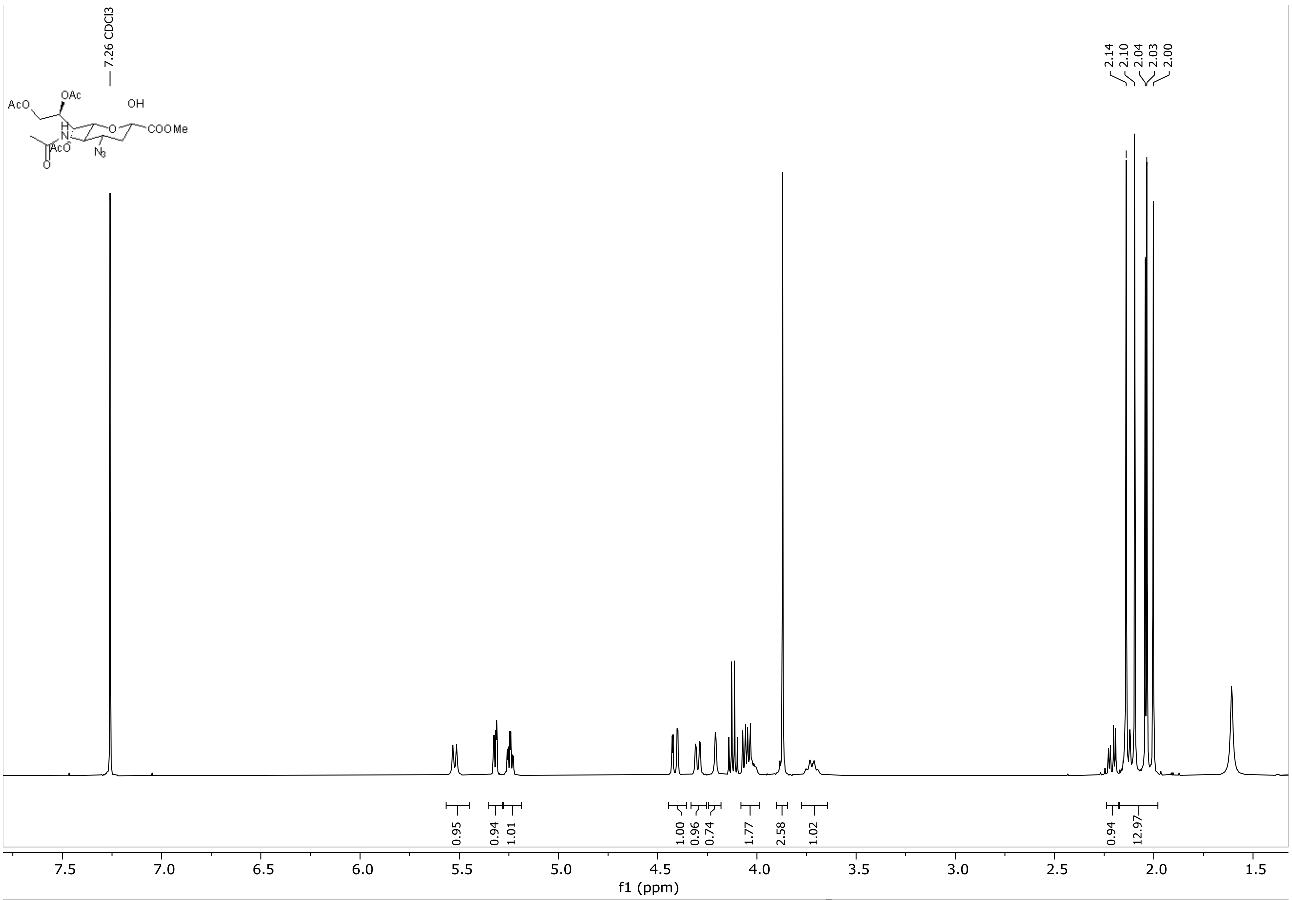

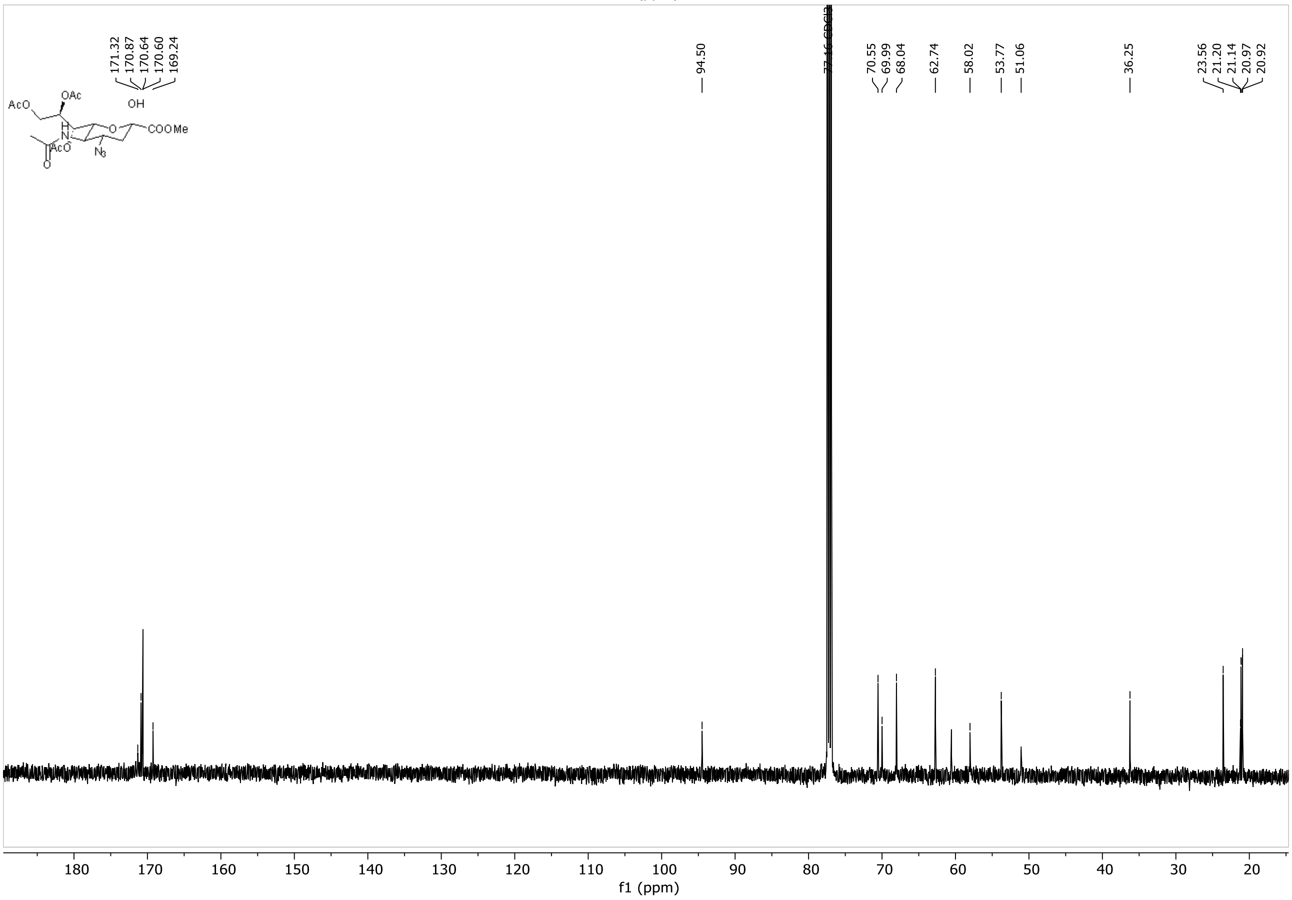

^{1}H- and ^{13}C-NMR spectra of compound 4g

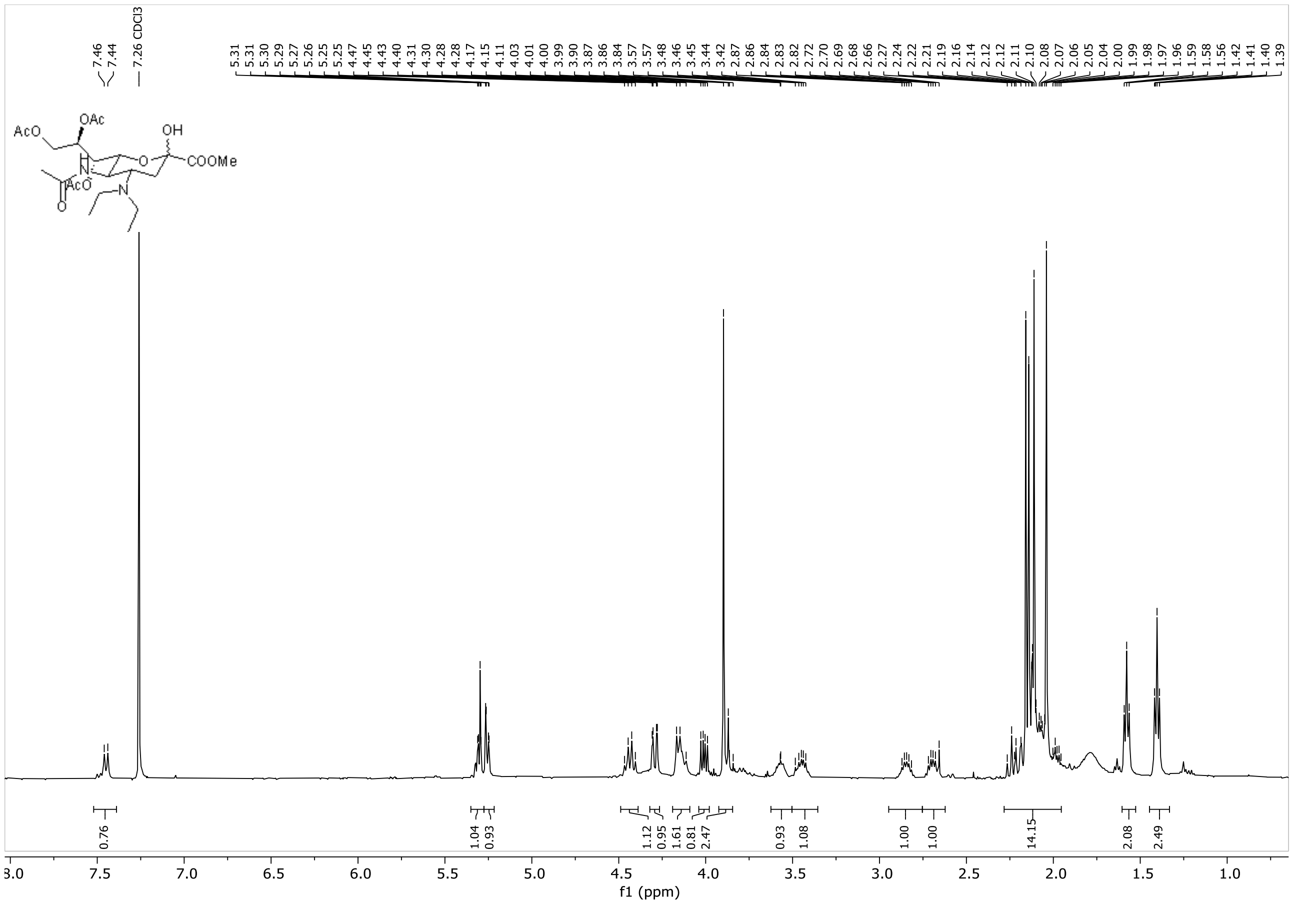

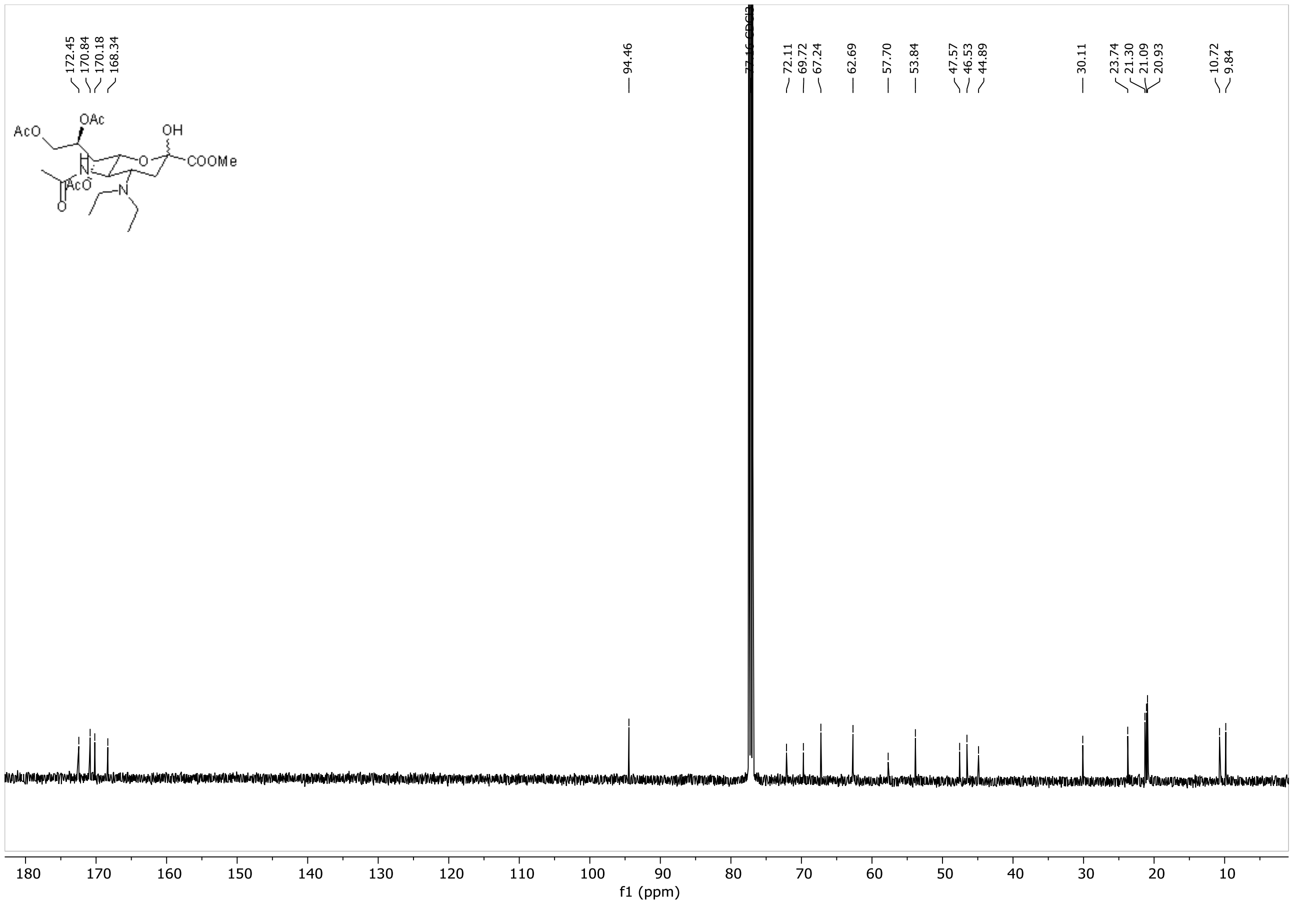

^{1}H- and ^{13}C-NMR spectra of compound 4h

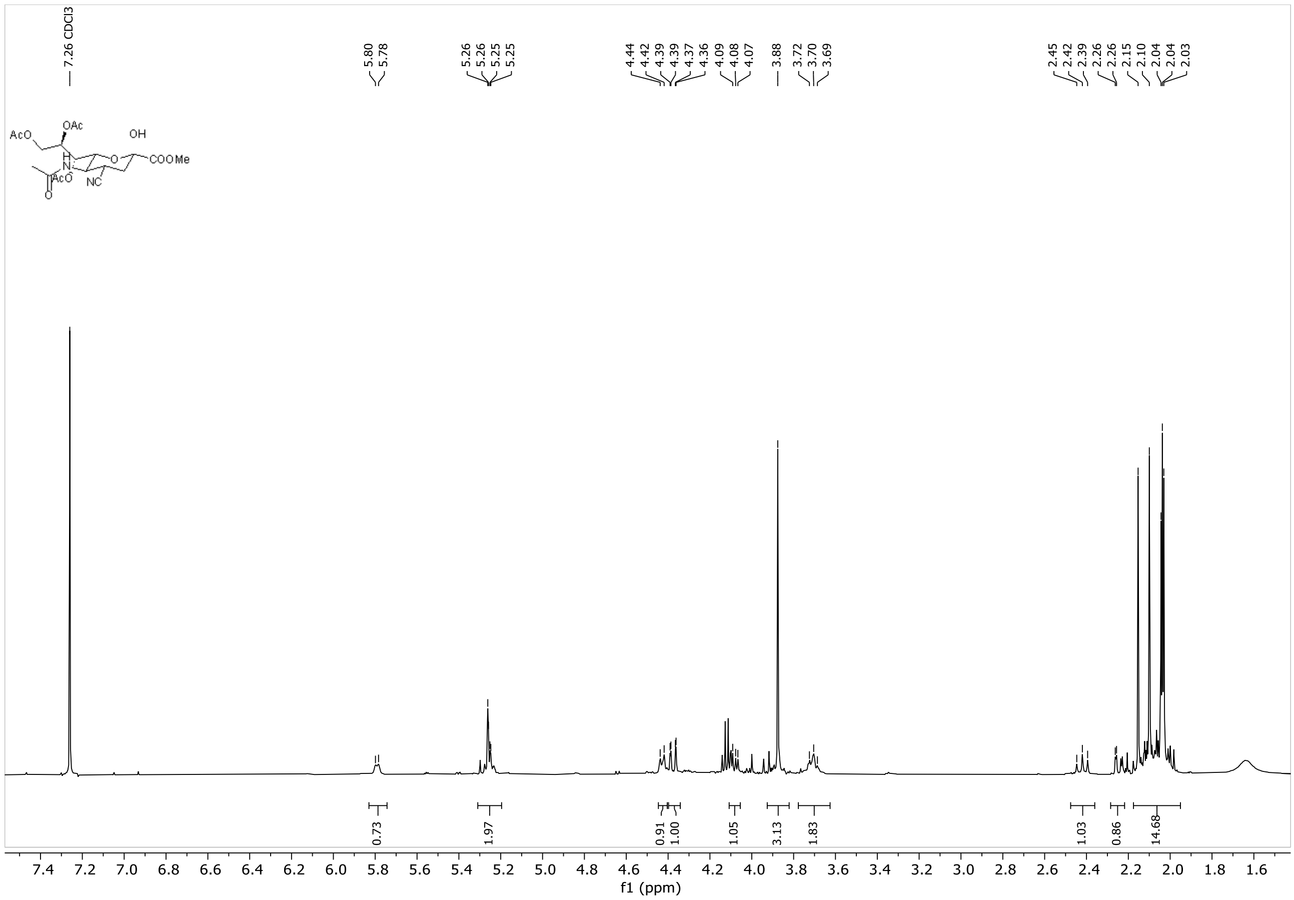

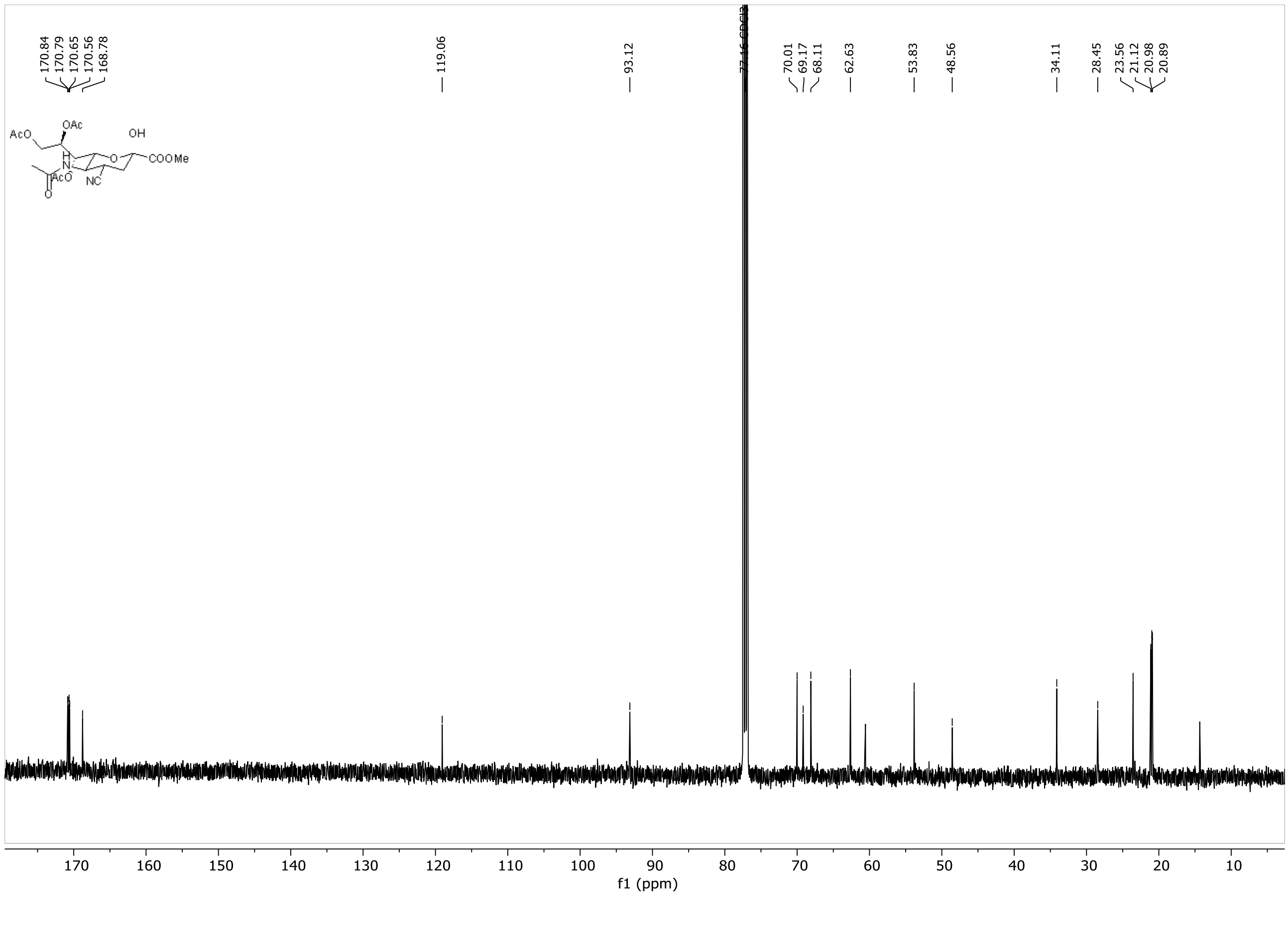

^{1}H- and ^{13}C-NMR spectra of compound 4i

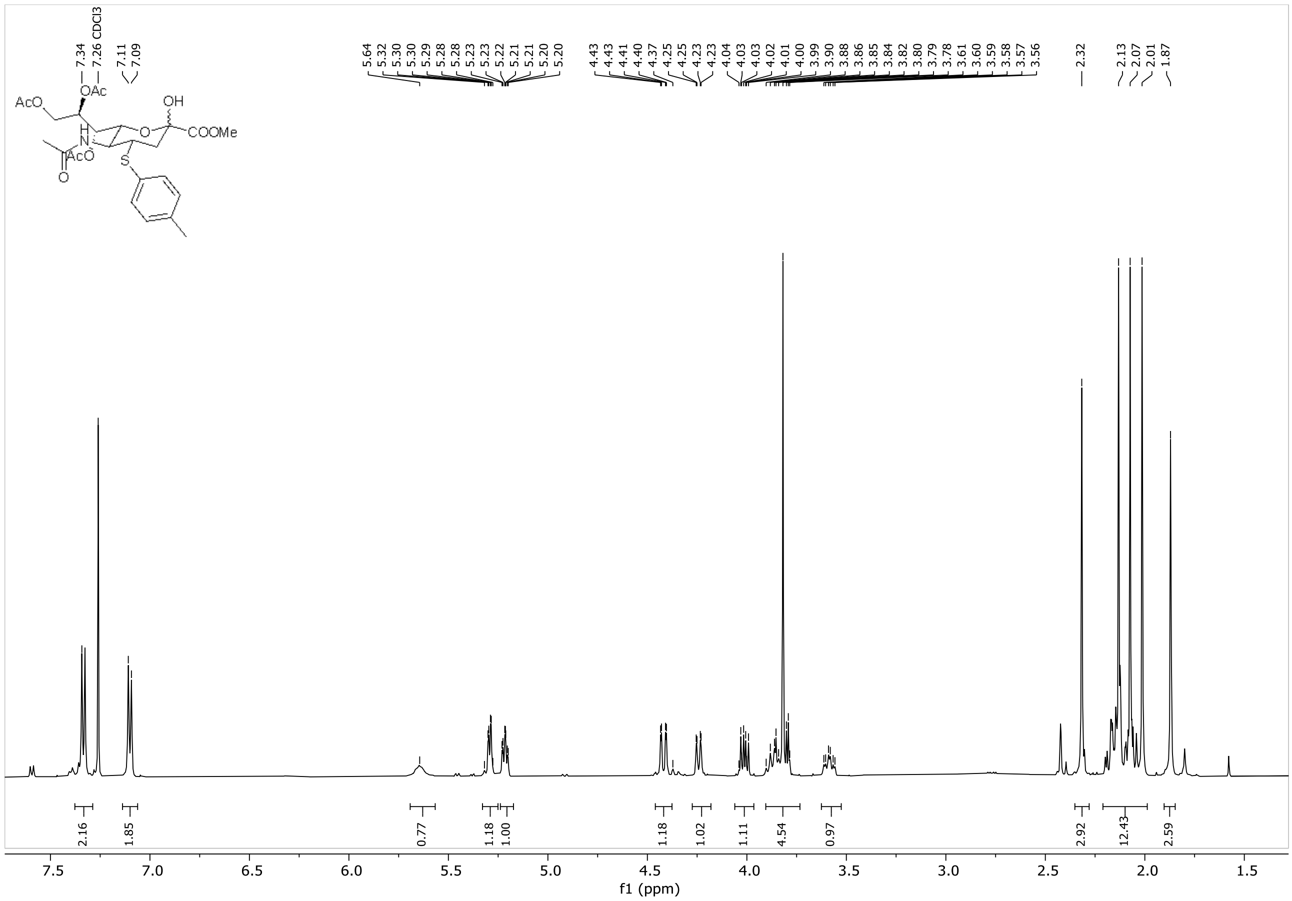

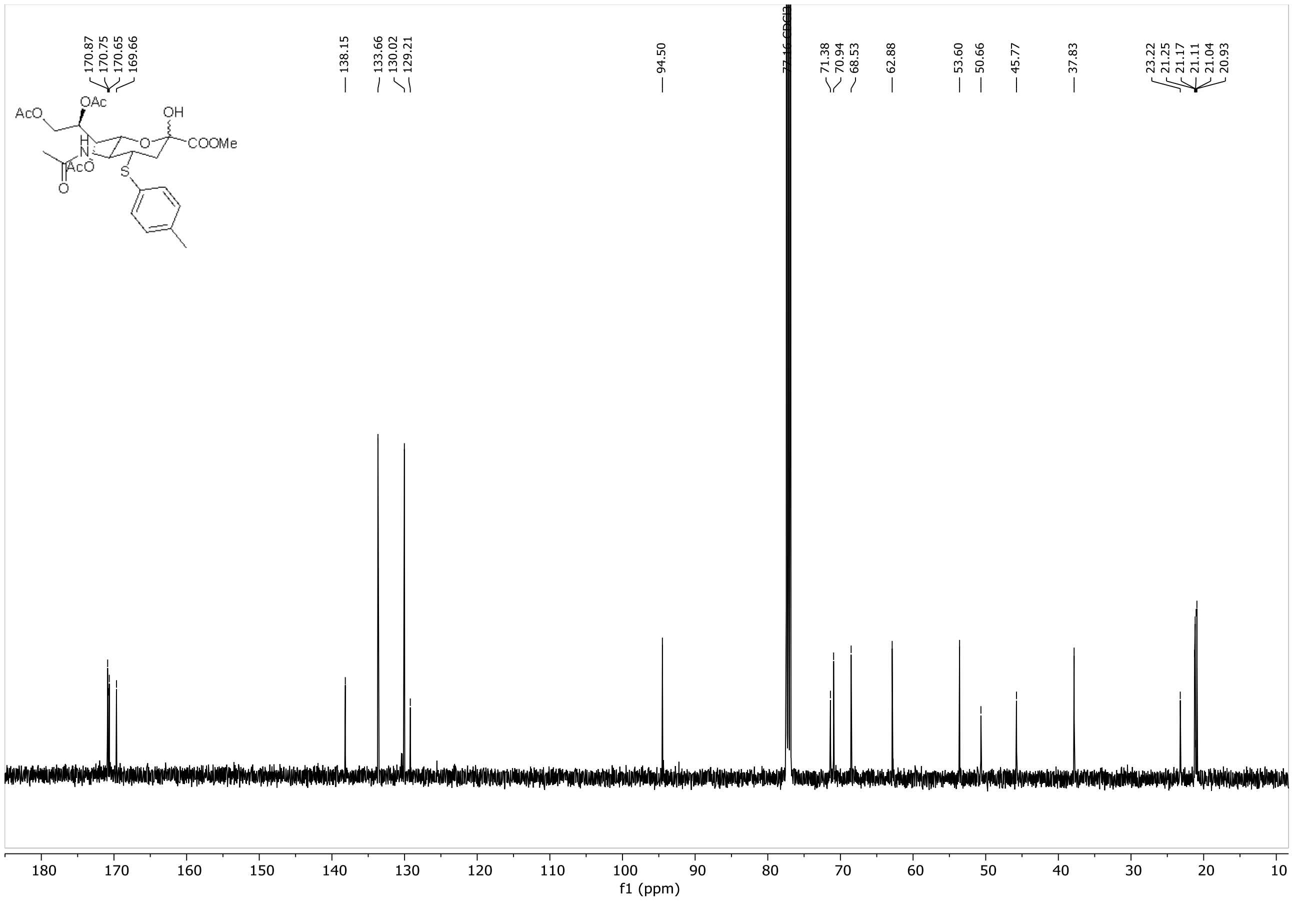

^{1}H- and ^{13}C-NMR spectra of compound 4l

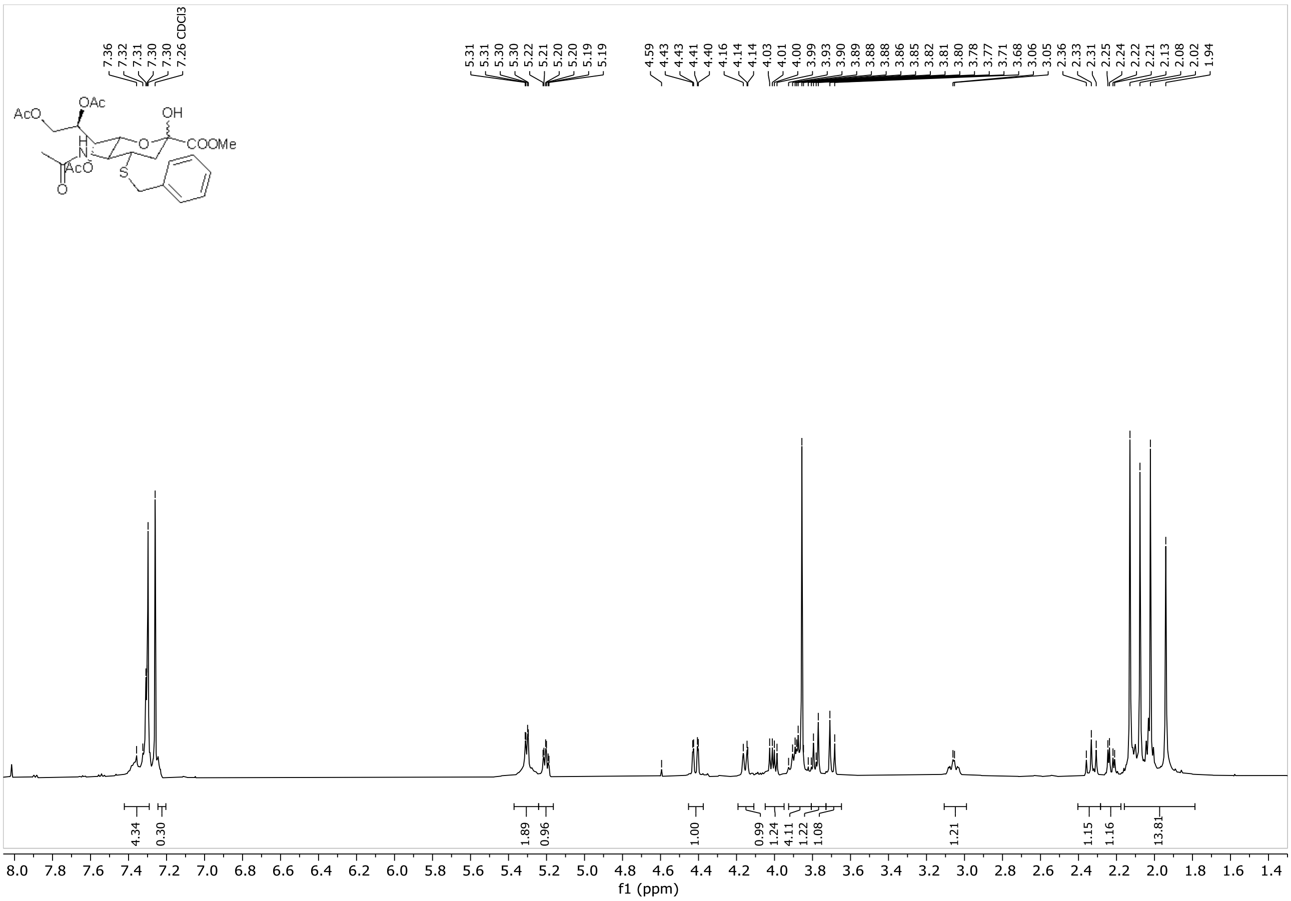

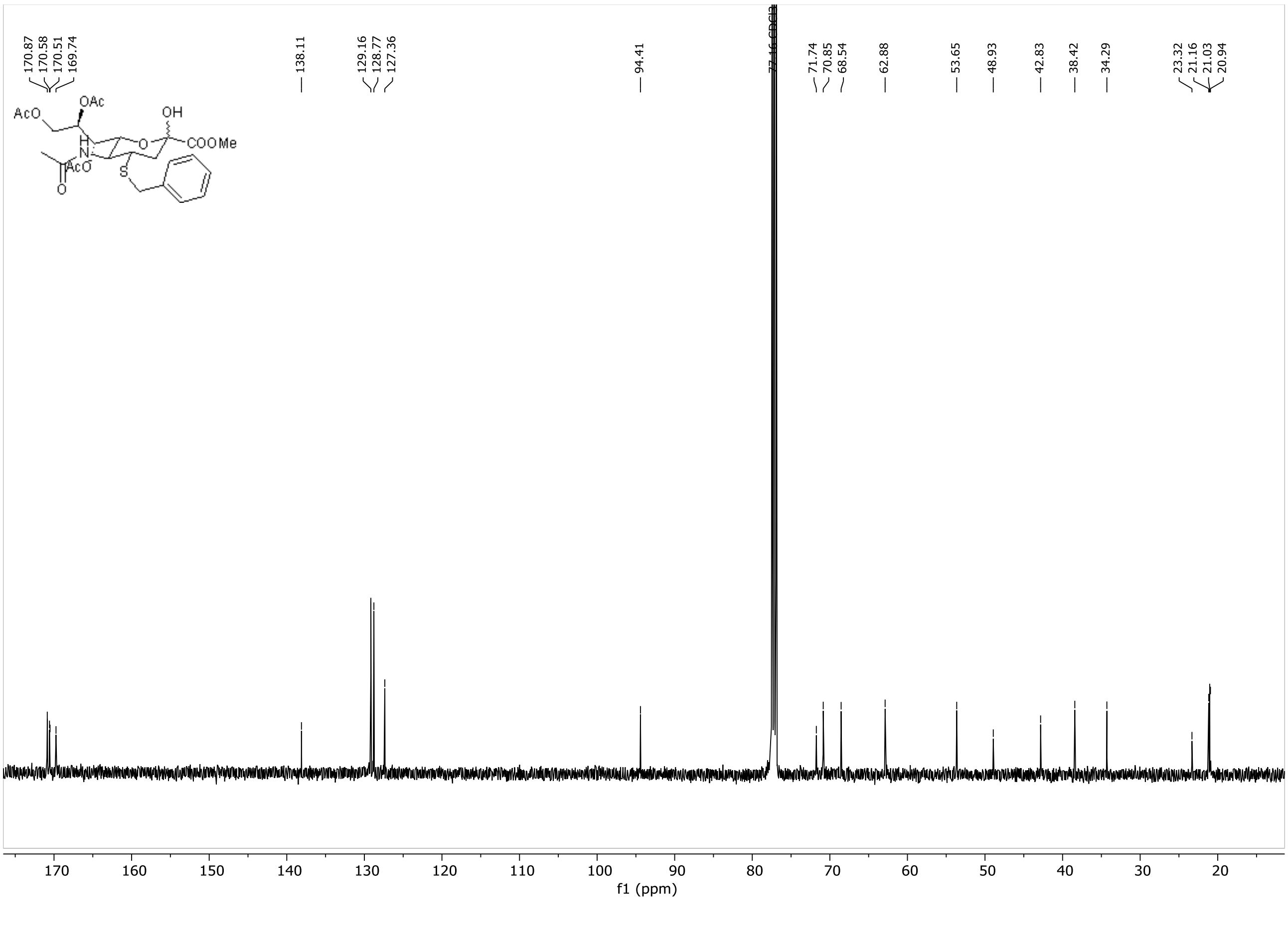

Supplement: RA-012-D2RA01576E-s001 [file RA-012-D2RA01576E-s001.pdf]
